# Neural Network Accelerated Investigation of the Dynamic Structure–Performance Relations of Electrochemical CO_2_ Reduction over SnO*_x_* Surfaces

**DOI:** 10.34133/research.0067

**Published:** 2023-03-14

**Authors:** Lulu Li, Zhi-Jian Zhao, Gong Zhang, Dongfang Cheng, Xin Chang, Xintong Yuan, Tuo Wang, Jinlong Gong

**Affiliations:** ^1^ School of Chemical Engineering and Technology, Key Laboratory for Green Chemical Technology of Ministry of Education, Tianjin University, Tianjin 300072, China.; ^2^ Collaborative Innovation Center for Chemical Science and Engineering (Tianjin), Tianjin 300072, China.; ^3^ National Industry-Education Platform of Energy Storage, Tianjin University, Tianjin 300072, China.; ^4^ Haihe Laboratory of Sustainable Chemical Transformations, Tianjin 300192, China.; ^5^ Joint School of National University of Singapore and Tianjin University, International Campus of Tianjin University, Binhai New City, Fuzhou 350207, China.

## Abstract

Heterogeneous catalysts, especially metal oxides, play a curial role in improving energy conversion efficiency and production of valuable chemicals. However, the surface structure at the atomic level and the nature of active sites are still ambiguous due to the dynamism of surface structure and difficulty in structure characterization under electrochemical conditions. This paper describes a strategy of the multiscale simulation to investigate the SnO*_x_* reduction process and to build a structure–performance relation of SnO*_x_* for CO_2_ electroreduction. Employing high-dimensional neural network potential accelerated molecular dynamics and stochastic surface walking global optimization, coupled with density functional theory calculations, we propose that SnO_2_ reduction is accompanied by surface reconstruction and charge density redistribution of active sites. A regulatory factor, the net charge, is identified to predict the adsorption capability for key intermediates on active sites. Systematic electronic analyses reveal the origin of the interaction between the adsorbates and the active sites. These findings uncover the quantitative correlation between electronic structure properties and the catalytic performance of SnO*_x_* so that Sn sites with moderate charge could achieve the optimally catalytic performance of the CO_2_ electroreduction to formate.

## Introduction

The ever-increasing CO_2_ concentration in the atmosphere has caused a series of problems including climate change and ocean acidification. Many countries have set the goal to achieve carbon neutrality, i.e., net zero CO_2_ emission, at about the middle of the 21st century. One of the potential ways to achieve carbon neutrality is CO_2_ electroreduction (CO_2_ER) by renewable electricity. It is also seen as a promising strategy to create valuable chemicals (CO, formate, and multicarbon products) due to its ambient operating conditions, environmental friendliness, and high reaction rates [1–5]. Among a variety of researched electrocatalysts, Sn-based electrocatalysts have been paid plenty of attention [6] over the past decades due to their high selectivity to the liquid product formate and the low cost of the catalysts themselves. Great endeavors have been devoted to rational catalyst design and synthesis, such as size optimization [7,8], component regulation [9–14], and electrolyte governing [15,16]. Nevertheless, Sn-based electrocatalysts for CO_2_ER still suffer from high overpotential, hydrogen evolution side reaction, and limited knowledge of active structures.

It is generally believed that cognition of the active sites and catalytic mechanism at the atomic level can provide theoretical guidance for the rational design of effective electrocatalysts. Numerous theoretical researches have been dedicated to exploring the intrinsic properties of Sn-based electrocatalysts for the improvement of energy efficiency and selectivity toward formate [17,18]. For instance, our previous work found that in-plane oxygen vacancy (O_v_) can regulate formate production and the post-reduction stage of SnO_2_ corresponds to the optimal state [19]. Furthermore, previous operando technique detection by Hu et al. has demonstrated that partially reduced tin oxide (SnO*_x_*) is the catalytically active species existing in Sn-based electrocatalysts [20–22] and the oxidation state can be attributed to tuning the electrocatalytic activity of CO_2_ER to formate [23]. It must be highlighted that the surface structure and corresponding mechanisms are only being investigated from a nearly static perspective by the present theoretical calculation and experiment. However, as a cathode catalyst, SnO*_x_* catalyzing CO_2_ER is also accompanied by obvious self-reduction, leading to the ambiguous perception of SnO*_x_* structural dynamics and site diversity [24]. The dynamism of catalyst structure determines the corresponding catalytic activity [25]. Moreover, the dynamic surface structure and site distribution bring great challenges to conventional local optimization methods at the static density functional theory (DFT) level [26]. To accurately provide atom-level structure information and pave the rational way to design effective and efficient SnO*_x_*-based catalysts, a comprehensive calculation method should be developed to systematically search for the dynamic reduction process and feature structure of metal oxide in large and long-term simulation scale.

This paper describes a combined molecular dynamics simulation with neural network fitted potential (MD-NN) [27,28] and first-principles calculations to explore the SnO_2_ reduction process, with SnO_2_(110) as the starting material because it is the thermodynamically most stable surface. The indentation of structural dynamics of SnO*_x_* followed by surface reconstruction and the mechanism exploration of CO_2_ER and hydrogen evolution reaction (HER) are probed. A simple regulatory factor, the net charge, is identified through analyzing model sites of SnO_2_(110) and can be utilized for predicting and regulating the product distribution of CO_2_ER and HER over SnO*_x_* catalysts. This discovery is also in line with the experimental measurements of the chronoamperometry test, x-ray diffraction (XRD), and quasi in situ x-ray photoelectron spectroscopy (XPS) semiquantitative analysis. Electronic analyses illustrate that the increased net charge in the process of SnO*_x_* reduction is filled in the antibonding states between key intermediates (HCOO*, *COOH, and H*) and active sites, and the simultaneous change of adsorption strength alters the rate-determining step and the theoretical limiting potential, thus leading to the fluctuation of activity and selectivity over dynamic SnO*_x_* surfaces. These results disclose a quantitative correlation between electronic structure properties and the catalytic performance of dynamic SnO*_x_* surfaces with diverse active sites. A promising strategy is proposed to investigate the adsorbate–surface interactions and the dynamic structure–performance relation in electrocatalysis.

## Results

### SnO_x_ surface reduction process

**Fig. 1.**
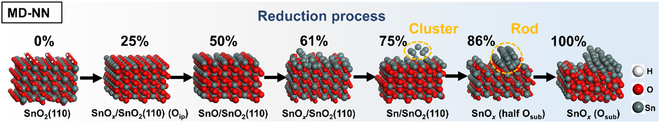
The MD-NN simulation of SnO_2_(110) reduction.

MD-NN was utilized to simulate the entire SnO_2_ surface reduction process, from SnO_2_(110) to metallic Sn, without any layers fixed (Fig. 1). To rule out the possibility of size dependence, model sizes were examined (Fig. S1 and Supplementary Materials). The reduction of SnO_2_(110) involves 3 different types of oxygen: bridging oxygen (O_bri_), in-plane oxygen (O_ip_), and first-layer subsurface oxygen (O_sub_) (Fig. S2). Specifically, we defined the reduction degree by the ratio of the lost oxygen atoms to the total of 3 kinds of oxygen atoms involved in the reduction process. The MD-NN simulation of 2 ns with the canonical ensemble (NVT) on each surface was conducted to find the equilibrium surface structure at 300 K. Radial distribution functions (Fig. S3) and potential trajectory (Fig. S4) were employed to determine whether the MD-NN was in equilibrium. The accuracy of NN potential is shown in Fig. S5, Tables S1 and S2, and Supplementary Materials. This strategy has successfully located the dynamic active site in our previous study about oxygen-derived Cu catalysts [29]. The reduction process includes the following several stages: the surface reduction: SnO_2_(110) (0%), SnO*_x_*/SnO_2_(110) (25%), SnO*_x_*/SnO_2_(110) (50%), SnO*_x_*/SnO_2_(110) (61%), and Sn/SnO_2_(110) (75%); deep reduction: SnO*_x_* (86%) (lost half O_sub_) and SnO*_x_* (100%) (lost all O_sub_), shown in Fig. 1.

The MD-NN simulation indicates the removal of O_bri_ and half O_ip_ did not cause remarkable surface reconstruction at the early reduction stage (from 0% to 50%). Subsequently, slight reconstruction occurred on the SnO*_x_*/SnO_2_(110) surface (61%, corresponding to the loss of all surface O_bri_ and O_ip_) (Fig. 1) and the Sn atoms tend to gather. Surface reconstruction becomes more obvious and Sn atoms aggregate to form the reconstructive structure from cluster to rod as the degree of SnO_2_ reduction increases (from 75% to 100% where the loss of O_sub_ occurs) through MD-NN. Additionally, the reduced structures agree with the in situ spherical aberration corrected scanning transmission electron microscopy (STEM) experimental study [30], which proves the reliability of our simulated structure. The surface Pourbaix diagram was also estimated on different reduced surfaces obtained from MD simulation, which shows that SnO_2_(110) (0%), SnO*_x_*/SnO_2_(110) (50%), SnO*_x_*/SnO_2_(110) (61%), SnO*_x_* (86%), and SnO*_x_* (100%) are thermodynamically stable under various potentials (Fig. S6). Furthermore, we also performed global optimization based on the stochastic surface walking (SSW) algorithm (Fig. S7). The optimized structures by both methods confirm the reconstruction feature (cluster or rod) during the reduction process, except for a few O evolutions from deeper metal oxide layers and aggregation with Sn atoms, indicating that the diffusion barrier of subsurface O to surface might cause SnO_2_ reduction to be sluggish during MD-NN simulation. The reliability of the SSW-NN method was also verified by increasing the number of steps (5,000) of the SSW-NN simulation and by comparison with the MD-NN results, as detailed in Fig. S8. Besides, simulated annealing confirmed the stability of the SnO*_x_* surfaces, which demonstrates that annealing may be utilized to surmount the energy barrier of MD-NN simulations and has a propensity to generate structures comparable to SSW-NN. However, the structure of active site on the surface level can still be maintained (Figs. S9 and S10).

### Electronic and geometric structure of the active sites

**Fig. 2.**
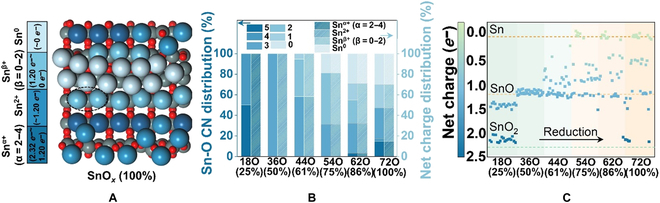
Electronic and geometric structure of active sites generated by the reduction progress. (A) The net charge distribution on SnO*_x_* surface (100%) (color code: blue: surface Sn; gray: Sn in bulk; red: oxygen). (B) Sn-O CN distribution of all surfaces during the reduction process. (C) The classification and proportion of the net charge as a function of reduction degree.

The surface reconstruction during the reduction process produces a variety of possible active sites with reduced Sn^δ+^. Taking the SnO*_x_*(100%) surface as the example in Fig. 2A with the most variety of active sites (other surfaces shown in Fig. S11), obvious surface reconstruction occurred on SnO*_x_*(100%), and the coordination number (CN) of Sn-O covers 0, 1, 2, 3, 4, and 5 (Fig. 2B). The Sn-O CN on all surfaces is counted in Fig. 2B in order to further differentiate these spots geometrically (the graphical example is shown in Fig. S12). Two key conclusions can be drawn from Fig. 2B: (a) the reduction of SnO*_x_* leads to a decreasing trend of Sn-O CN, and (b) the surface reconstruction makes the CN of Sn-O distribution more dispersed, resulting in a variety of Sn active sites.

The loss of oxygen in SnO*_x_* leads to the decrease of the oxidation states of surface Sn (Fig. 2A). Therefore, the statistics of the Sn oxidation state distribution on all reduced surfaces were also calculated (Fig. 2B and C). We use the net charges for the stoichiometric ratio SnO_2_, SnO, and Sn as the reference to estimate the oxidation state of Sn. As shown in Fig. 2C, the surface site of Sn^δ+^ can be approximately categorized by the following types: Sn^α+^ (α = 2 to 4, ~2.32 *e^−^* to ~1.20 *e^−^*), Sn^2+^ (~1.20 *e^−^*), Sn^β+^ (β = 0 to 2, ~1.20 *e^−^* to ~0 *e^−^*), and Sn^0^ (~0 *e^−^*). The proportion of different types of active sites as a function of reduction degree is illustrated in Fig. 2B, demonstrating that the overall values of the net charge show an upward trend with the increased degree of SnO*_x_* reduction. The dispersed distribution occurs at the post-reduction stages, mainly from Sn^β+^ to Sn^0^, leading to multiple types of active sites. It can be concluded that the trends of the net charge are consistent with the CN of Sn-O, which means the dynamism in geometric structure leads to the redistribution of electron density.

### Structure–performance relations

To explore the effect of surface reduction and reconstruction on CO_2_ER and side reaction (HER) mechanisms, possible pathways and key intermediates were taken into consideration to explore their mechanisms and catalytic activity.

**Fig. 3.**
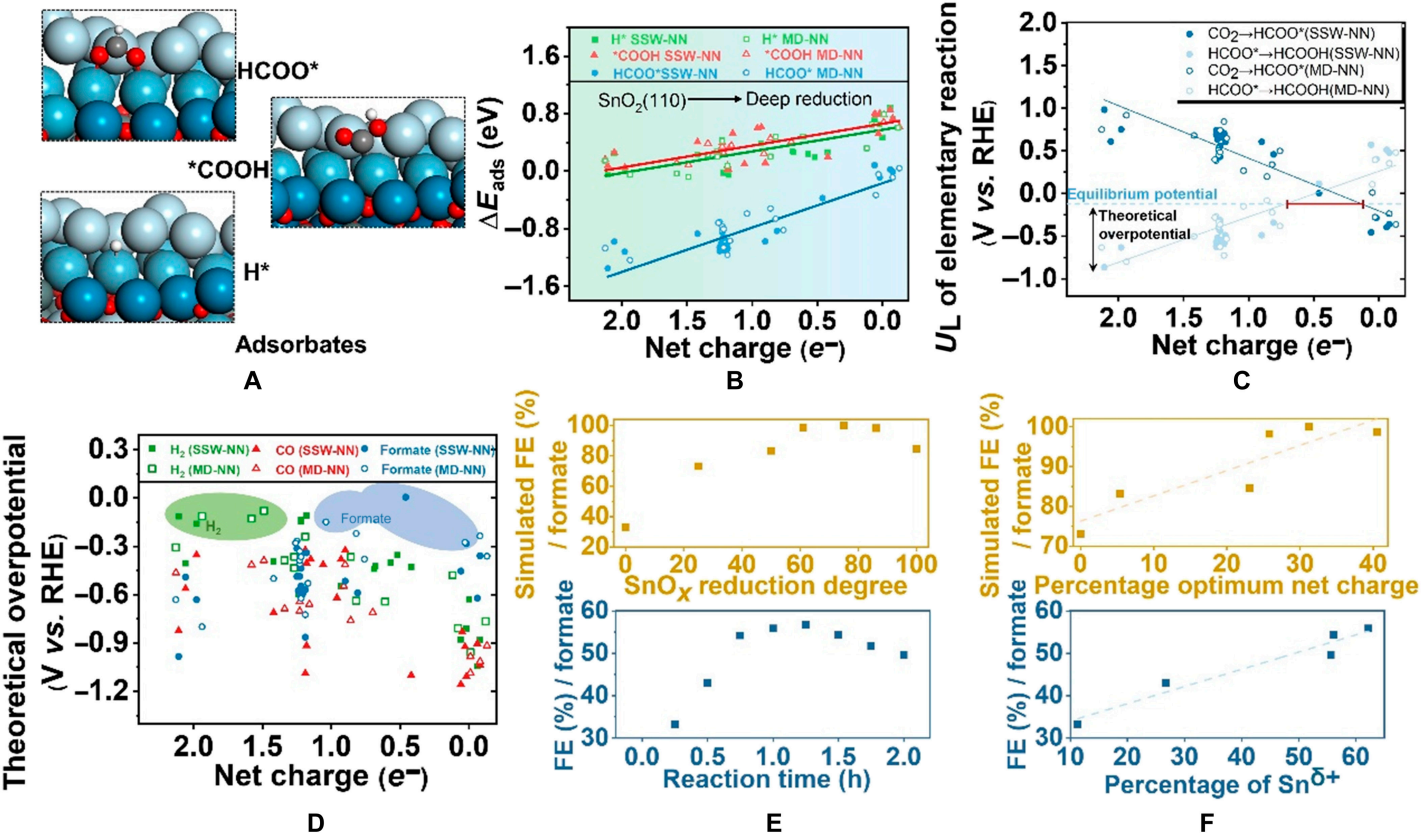
The effect of surface reduction on CO_2_ER and HER. (A) Adsorption configuration diagram of H*, *COOH, and HCOO* on SnO*_x_* surface. (B) The correlation of ∆*E*_H*_, ∆*E*_COOH*_, and ∆*E*_HCOO*_ and the net charge on different surfaces (solid shapes: MD-NN simulated surfaces; hollow shapes: SSW-NN optimized surfaces). (C) Limiting potentials (*U*_L_) as a function of net charge for the 2 elementary reactions of CO_2_ER to formate at Sn active sites. (D) The theoretical overpotential for CO_2_ER and HER. (E) The FE of formate, CO, and H_2_ on different SnO*_x_* surfaces (top: theoretical calculation) and different reaction times (bottom: experiment). (F) The correlation of FE and percentage of Sn^δ+^ (top: theoretical calculation; bottom: experiment).

Previous studies have revealed that the selectivity can be determined by the competitive adsorption of the 3 initial hydrogenation intermediates, namely, HCOO* for CO_2_ER to formate, *COOH for CO_2_ER to CO, and H* for HER to H2. It should be added that for the attribution of the reaction intermediates of formate and CO, the current reasonable interpretation is that the C-bound mode (*COOH, carbophilic) corresponds to the production of CO and the O-bound mode (HCOO*, oxidophilic) corresponds to the production of formate [11,31] (see details in the Supplementary Materials). The adsorption configuration is shown in Fig. 3A. The adsorption strength of the key intermediates of CO_2_ER, as well as HER, was calculated at the DFT level and the corresponding net charge of active sites was also related in Fig. 3B. Besides, the effect of dispersion-corrected functionals on adsorption energies was tested in Fig. S13 and Table S3. Each adsorbed species and the net charge of active sites are taken from no less than 40 sets of data among the 210 sites that occurred during the whole reduction process. Interestingly, we discovered that ∆*E*_H*_, ∆E_*COOH_, and ∆*E*_HCOO*_ can be described as a function of the net charge of Sn active sites. These results can lead to the conclusion that the net charge most likely plays a direct and important role in regulating the adsorption strength of all 3 intermediate species (∆*E*_H*_ = –0.30 *e^−^* + 0.58, ∆E_*COOH_ = –0.31 *e^−^* + 0.66, and ∆*E*_HCOO*_ = –0.62 *e^−^* – 0.17)

Further research was done on the effect of adsorption energy (∆*E*_ads_) on the trends of the CO_2_ER and HER (H_2_, CO, and formate) activities. It is commonly accepted that the theoretical overpotential that ensures each elementary step of a reaction becomes exergonic [32] can be used to estimate the reaction activities theoretically [33]. Besides, CO_2_ER to formate and CO, as well as HER, include only 2 elementary reaction steps. Therefore, the theoretical overpotential can be obtained from the free energies of intermediates (∆*G*_ads_), or the difference between the equilibrium potential and ∆*G*_ads_. The correction of ∆*G*_ads_ from ∆*E*_ads_ is provided in the Supplementary Materials and Table S4. In this way, the theoretical overpotential of different products, such as CO_2_ER to formate in Fig. 3C, can be detected based on the relations of net charge and ∆*E*_ads_. The results show that the theoretical overpotential depends on the second reaction step when the net charge is large, due to the strong adsorption of HCOO*. The adsorption capacity of HCOO* decreases as the net charge decreases, at which point the potential-determining step shifts to the first one; in this way, a volcano-shaped activity of CO_2_ER to formate emerges and the theoretical optimal net charge range is approximately from 0.77 to 0.08 *e^−^*. The trends of CO and H_2_ are also calculated, as shown in Fig. S14. The theoretical overpotential of 3 products is summarized in Fig. 3D, demonstrating that when the value of net charge for Sn sites is high, strong binding of HCOO* at Sn sites leads to a highly energy-consuming step of HCOO* to formate in thermodynamics. Besides, excessively weak *COOH adsorption results in a high overpotential of CO. Nevertheless, the adsorption strength of H* is optimal for HER, indicating that the generation of H_2_ is preferable to that of formate and CO (green area in Fig. 3D). As the net charge decreased (blue area), the weakening of HCOO* adsorption capability could make the generation of HCOO* from CO_2_ become the potential-determining step and the theoretical overpotential of formate becomes the lowest. It leads to the priority of the target product and inhibition of H_2_. This means that the related catalytic selectivity is likewise impacted by the dispersion of the net charge distribution brought on surface reduction and reconstruction. Specifically, we can draw the theoretical overpotential as a function of net charge and then directly derive the relevant catalytic activity and selectivity from those profiles owing to the linear relations between the net charge of active sites and the adsorption energy of key intermediates. Thus, the net charge may be considered as a streamlined descriptor to forecast the dynamic structure–performance relations.

Next, we further predicted the theoretical catalytic performance on various SnO*_x_* surfaces and directly validated it with electrochemical experiment, based on the redistribution of active sites induced by SnO*_x_* reduction and surface reconstruction. As was stated earlier, the only input necessary for DFT calculations is the net charge of Sn sites. Thus, the fitted ∆*E*_ads_ directly leads to the theoretical overpotential after the correction of free energy. The theoretical Faradaic efficiency (FE) of CO_2_ER to formate and CO, as well as HER, can be simulated by substituting the prefactors, obtained by fitting the experimental value by –1.2 V vs. reversible hydrogen electrode (RHE) (Fig. 3E and Fig. S15a, details in the Supplementary Materials) [34,35]. The corresponding net charge and the number of surface atoms (*N*_surf_) exposed to the different reduced degree surfaces are counted 5 times during the MD equilibrium stage. Figure 3E (top) shows that the average FE of formate increases first and then decreases with the reduction of SnO_2_(110) and shows the volcanic peak in a moderate reduction stage (61% and 75% of SnO*_x_* surfaces). To verify the predicted trends of the CO_2_ER on SnO*_x_*, we prepared electrodes using commercial SnO_2_ (50 to 70 nm) loaded onto commercial gas diffusion layers (GDLs) to represent the SnO*_x_* model used in the simulations. The FE of generated formate is quantified by the chronoamperometry test at −1.2 V RHE. The results are shown in Fig. 3E (bottom), which demonstrates that the formate FE trend is consistent with the theoretical simulation. The XRD pattern in Fig. S16 does not show the phase change of SnO_2_, indicating the stability of the SnO_2_ bulk phase structure during the reaction time range of 0.0 to 2.0 h. The chemical state of SnO*_x_* is examined by quasi in situ XPS studies [36,37]. The binding energy of Sn^δ+^ (chemical state between 0 and 4+) varies linearly with the reduction time as shown in Fig. S18c, indicating that the reduction time can be used to express the surface reduction degree of SnO*_x_*. The percentage of Sn^δ+^ is also detected quantitatively by theoretical statistics and quasi in situ XPS studies in Fig. 3F and Fig. S18 to correlate with the formate FE trend. Both theoretical calculations and experimental results show that there is a direct linear positive correlation between the performance of the main product formate and the proportion of the partially reduced Sn^δ+^. More precisely, the chemical state of Sn is between 0 and 2+ as illustrated in Fig. 3F. Besides, the operando Raman and x-ray absorption spectroscopy (XAS) methods [21] monitoring the structural changes of SnO*_x_* also provide evidence that the Sn^δ+^ state during CO_2_ER corresponds to the optimal formate production.

### The origin of adsorbate–surface interaction

To gain deep insights into the specific impact of the net charge on the catalytic performance of CO_2_ER as well as HER, electronic structures at the DFT level were further conducted.

**Fig. 4.**
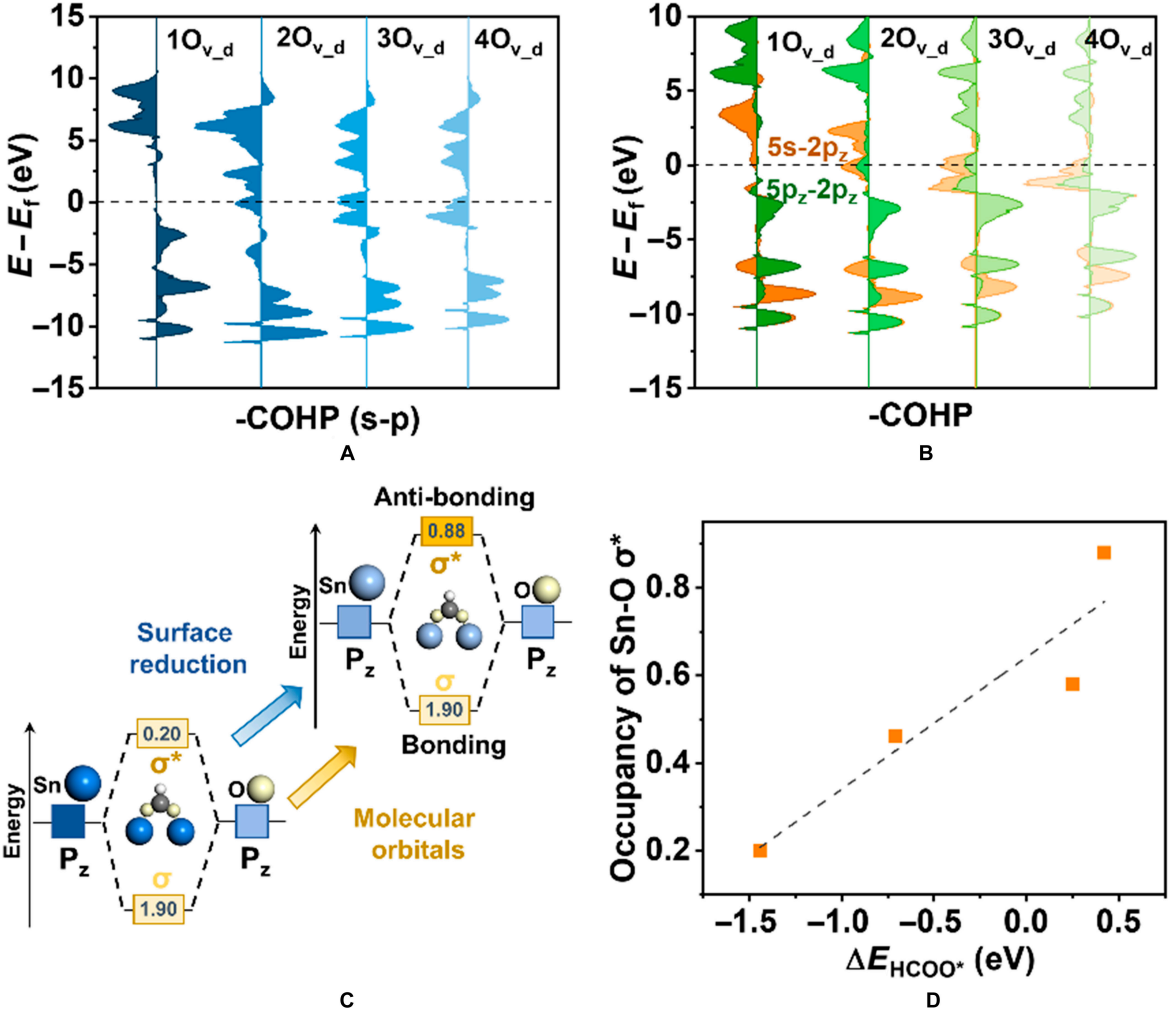
Electronic structures of adsorbates and active sites. (A and B) The pCOHP between the metal center of Sn with different degrees of reduction and O atom of HCOO* (A: Sn s orbital and O p orbital; B: Sn 5s orbital and O 2p_z_ orbital). (C) Scheme of molecular orbital between HCOO* and Sn site. (D) The correlation of ∆*E*_HCOO*_ with occupancy of Sn-O σ*.

What needs to be illustrated here is that we design a representative model by changing the CN of Sn-O on the SnO_2_(110) surface from a geometry perspective to construct distinct and definite active sites (Fig. S19). Additionally, other facets such as SnO_2_(101) and SnO_2_(100) are also in line with the universal relation of the net charge and ∆*E*_ads_ (Fig. S20). The linear correlations between ∆*E*_ads_ and the integrated crystal orbital Hamilton population (iCOHP) can be quantitatively explained initially such that the adsorption strength is directly related to the interaction between the adsorbates and Sn active sites (Fig. S21 and Tables S5 to S7). The projected COHP (pCOHP) of Sn-O (HCOO*) bonding (Fig. 4A and Fig. S22) shows that the antibonding orbitals of s–p interaction move down with the increase in reduction degree, from one O_v_ (1O_v_d_) to 4O_v_d_. For details, see Fig. 4B and Fig. S23, where the pCOHP of s–p orbitals demonstrates that the antibonding states close to and below the Fermi level primarily stem from Sn 5s as well as Sn 5p_z_, which interacted with O 2p_z_ atomic orbitals. This means that Sn 5s and 5p_z_ atomic orbitals essentially contributed to the enhanced charge density of Sn sites. Meanwhile, the pCOHP analyses on *COOH and H* with Sn sites reveal that the weakening of Sn-C and Sn-H interaction are also due to the increase in the filling degree of the corresponding antibonding, namely, Sn 5s with H 1s and Sn 5s with C 2p_z_ (Figs. S24 and S25). Therefore, the key intermediates of CO_2_ER to C1 products and HER on the active sites can be modulated by the occupation of antibonding orbital near the Fermi level, which is related directly to the geometrical SnO_2_ reduction together with the net charge of Sn sites.

The chemical bonding configuration of active sites and adsorbates was conducted by periodic natural bond orbital (pNBO) (Tables S8 and S9). The σ* bond occupancy of Sn-O in HCOO* is 0.20 *e^−^* with 1O_v_d_ and rose to 0.88 *e^−^* with 4O_v_d_ in Fig. 4C. This increasing trend of σ* bond occupancy in Fig. 4D is also consistent with the analyses of antibonding from iCOHP, indicating that the adsorption interaction weakens as SnO_2_(110) is reduced. By evaluating the hybridization of σ* (1O_v_d_: 88.92 %, 2O_v_d_: 87.55 %, 3O_v_d_: 90.73 %, and 4O_v_d_: 87.36 %, Table S8), it can be shown that the σ* orbital makes up the major contribution from Sn atoms. The pNBO analyses of Sn-C in *COOH also demonstrate that the σ* bond occupancy displays a positive relation with the concentration of O_v_ (Table S9), further confirming that an increase in the net charge caused by SnO_2_ reduction affects the antibonding filling, thus weakening the interaction strength between active sites and adsorbates.

## Discussion

**Fig. 5.**
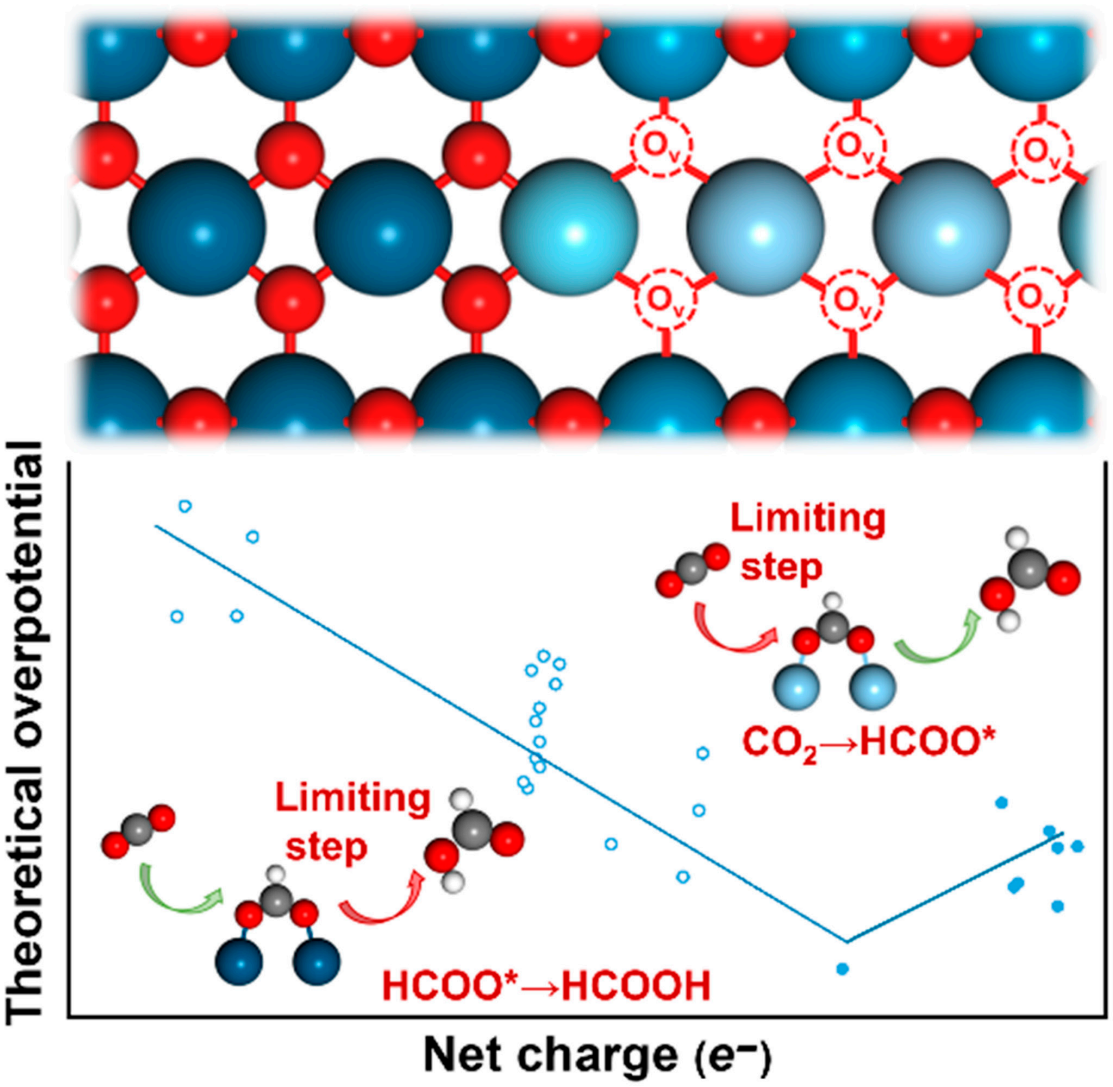
The origin of electronic interaction and bonding between the active site Sn of different reduction degrees and HCOO*. The interaction leads to the change of limiting step and potential of CO_2_ER to formate.

Based on the above analyses, Fig. 5 outlines that SnO_2_ reduction in geometry leads to the increase in the charge density of Sn sites essentially. In particular, when interacting with the adsorbate (HCOO*), the increased charge in 5s and 5p_z_ orbitals injects into the antibonding formed with O 2p_z_ orbital, which weakens the adsorption capacity of SnO*_x_* and accounts for the differing adsorption strength of HCOO* on SnO_2_ with various O_v_. It can be summarized that when the degree of SnO*_x_* reduction is low, the strong binding of HCOO* leads to the step of HCOO* to formate becoming the theoretical limiting step. As the degree of SnO*_x_* reduction deepens, the increased net charge of Sn site is injected into the antibonding orbital between Sn-O (HCOO*), remarkably weakening the binding ability of HCOO*, thus gradually changing the theoretical limiting step to the HCOO* formation step. Therefore, we can infer that the net charge of Sn sites is a key regulatory electronic factor that governs the interaction ability with adsorbates. Tuning the net charge of the active site can be regarded as an efficient strategy to promote the catalytic performance of SnO_2_ for CO_2_ER to formate theoretically.

In summary, this paper put forward a method that combined NN potential accelerated MD, global optimization, and first-principles calculations to simulate the dynamic reduction process of SnO_2_ and investigate the nature of Sn active sites as well as the electronic structure effect on the activity and selectivity of CO_2_ER and HER. These results demonstrate that the catalytic performance of SnO*_x_* changes with the surface reconstruction geometrically together with the charge distribution of Sn active sites. Moreover, a quantitative and dynamic structure–performance relation between the net charge and the ∆*E*_ads_ was fit. Furthermore, by exploiting this relation, we can statistically estimate the proportion of different sites obtained from MD-NN simulation and thus invert the apparent catalytic performance of CO_2_ER over different SnO*_x_* surfaces, which is also found to be consistent with the experimental test. The model sites reveal that the nature of Sn active sites is responsible for the catalytic performance; that is, the antibonding occupation is enhanced with the increased net charge of Sn active sites, leading to the variation in the interaction between Sn sites with the adsorbates. Finally, a theoretical guideline for the improvement of formate selectivity is proposed: The Sn active sites with a moderate amount of net charge, which can be achieved by tuning the reduction degree of SnO*_x_*, performs an excellent catalytic activity of CO_2_ER. It should be noted that as an electrocatalytic system, the electrical double layer, the electrolyte solution, and the dissolution/redeposition process of electrodes should be considered. However, the force fields currently developed to simulate electrochemical models are still very crude, and the simulation time of MD is limited. We are also currently conducting related research, including potential fitting and application of solid–liquid interface simulation. In this study, the MD-NN simulation method is mainly used to figure out the surface structure and the feature sites of SnO*_x_*. These explorations illustrate how a versatile and dynamic simulation approach can explore the key regulatory factor for CO_2_ER and provide rational guidance to promote the catalytic performance of metal oxide in electrocatalysis.

## Materials and Methods

### MD-NN and SSW-NN simulations

The large-scale atomistic simulation with neural network potential (LASP) program developed by Huang et al. [38] (http://lasphub.com/%23/lasp/laspHome) was used to resolve the complex oxide structures based on global structure and molecular dynamics simulation (SSW-NN and MD-NN) methods. The SnO.pot and SnOH.pot trained by NN-based SSW PES exploration [39] were adopted over the reduction process of SnO_2_ to search the stable structures at different reduced stages. The global datasets generated from the high-accuracy first-principles calculations contain 17,343 and 40,143 structures for SnO and SnOH systems, and further details are listed in Tables S1 and S2. The root-mean-square errors of SnO.pot and SnOH.pot potentials are 9.018 and 6.264 meV/atom, respectively. The comparison of NN potential predicted energy and that from DFT calculation of H* adsorption energy (active site: O and Sn) are shown in Fig. S5; the results indicate that the accuracy of NN potential can reach the level of DFT calculation. In order to explore the globally stable and dynamically reduced structures, SSW-NN and MD-NN methods are carried out respectively to optimize the reduction process of SnO_2_.

As for global optimization, SSW step was set to 2,000 for SSW-NN. As for MD-NN simulations, 2 ns of MD-NN simulation with NVT ensemble at 300 K was carried out for each structure with different reduced degrees [29]. Throughout these processes, no layer of the model system is fixed. After the simulation of the whole reduction process, all the low-energy structure candidates from the SSW-NN and MD-NN exploration and related calculations of CO_2_ER and HER reaction mechanisms were executed by plane-wave DFT next. The O atoms deeper than the first-layer subsurface oxygen were not considered in the reduction process due to deeper O atoms having little effect on the surface Sn.

### DFT calculations

DFT calculations were carried out using the Vienna ab initio Simulation Package (VASP) code [40,41] in conjunction with the computational hydrogen electrode model [42]. We adopted the generalized gradient approximation in the form of the Perdew-Burke-Ernzerhof (PBE) functional and projector augmented wave pseudopotentials [40] to describe the electron exchange and correlation effects [43]. A cutoff energy of 400 eV and an atomic force convergence of 0.02 eV/Å were employed. Four-layer (6×3) periodic slabs of the SnO*_x_*/SnO_2_(110) surfaces were modeled, where the 2 bottom layers were kept fixed, and the Monkhorst-Pack k-mesh of 2×2×1 was adopted for integrations over the Brillouin zone for all systems. In addition, the spin polarization scheme was adopted for all calculations. A vacuum space with at least 15 Å was set between the slabs to the periodic images in the vertical direction. As for the model simulation, a (4×2) slab with 4 layers is constructed. The Monkhorst-Pack k-mesh was set to 3×3×1 and the vacuum space was also at least 15 Å.

The free energy for each intermediate state is calculated as *G* = *E*_DFT_ + ZPE + δH_0_ − *TS*, where *E*_DFT_ is the DFT total energy, ZPE is the zero point vibrational energy, δH_0_ is the integrated heat capacity, *T* is the reaction temperature, and *S* is the entropy. For gas and liquid phase species, the δH_0_ and entropy *S* at 298.15 K were obtained from Dean [44]. For adsorbates, the entropy was calculated with the harmonic oscillator approximation (see Table S4). The solvation corrections of the water–solid surface interface are included in this work: –0.5 eV for OH*, –0.2 eV for COOH*, and –0.25 eV for HCOO* [42,45]. The theoretical *U*_L_ is numerically equal to the negative value of the biggest Gibbs free energy change of CO_2_ER to formate or CO and HER (*E* = –Δ*G*_max_/*zF*, where *z* is the number of electrons transferred, *F* is the Faradaic constant, and *E* is the limiting potential). The computational details can be found in the Supplementary Materials.

The COHP analysis was conducted through the Lobster 3.2.0 code, upon a transformation of the (plane) wave functions from VASP into a localized basis set [46,47]. Besides, the pNBO formalism developed by Dunnington and Schmidt [48] was also applied using VASP software to analyze surface chemical bonding via natural bond orbitals.

### The SnO_2_ electrode preparation

The obtained SnO_2_ was airbrushed onto the commercial carbon-based GDLs at an approximate loading of 0.9 mg/cm^2^, measured through weighing GDLs before and after airbrushing. The catalyst ink was prepared by dispersing 200 mg of SnO_2_ and 50 μl of Nafion solution (Sigma-Aldrich) in 750 μl of isopropyl alcohol and 250 μl of ultra-purity water and sonicated for 1 h before airbrushing (H-SET, Paasche).

### Electrochemical test

All electrochemical experiments were conducted in a gas-tight H-type electrochemical cell machined from poly(methylmethacrylate) (PMMA). The cell was thoroughly rinsed with ultra-purity water prior to all experimentation. The working and counter electrodes were parallel and separated by a bipolar membrane (fumasep, FBM). Gas dispersion frits were incorporated into both electrode chambers to provide ample gas-electrolyte mixing. The exposed geometric surface area of the working electrode (the SnO_2_ electrodes) was 1 cm^2^ and the electrolyte volume of each electrode chamber was 10 ml. The counter electrode was an IrRu alloy deposited Ti mesh (5 cm^2^). The working electrode potential was referenced against a leak-free Ag/AgCl electrode (Harvard Apparatus) that was calibrated against a homemade standard hydrogen electrode. Metallic impurities in the as-prepared electrolyte (0.1 M KHCO_3_) were removed before electrolysis by chelating the solution with Chelex 100 (Na form, Sigma Aldrich). Both electrode chambers were sparged with CO_2_ at a rate of 5 sccm for at least 30 min prior to and throughout the duration of all electrochemical measurements. Upon saturation with CO_2_, the pH of the electrolyte was 6.8, which was maintained throughout the duration of chronoamperometry (PGSTAT204, Autolab).

During electrolysis, gas products were quantified using an online gas chromatography system (GC7890B, Agilent Technologies, Inc.). The thermal conductivity detector was connected to a MolSieve 5A packed column (Agilent Technologies, Inc.) to detect H_2_, O_2_, and N_2_, and a back flame ionization detector (FID) was connected to a Porapak Q packed column (Agilent Technologies, Inc.) to detect CO. A methanizer was installed to enable the back FID to detect CO with 1,000 times higher sensitivity. A front FID was connected to an HP-PLOT Al_2_O_3_ capillary column (Agilent Technologies, Inc.) to detect hydrocarbons (C1 to C3). Ar was used as the carrier gas. After passing through the reactor, the gas was allowed to flow directly into the gas sampling loop of the gas chromatography for online gaseous product analysis. The signal response was calibrated by analyzing a series of standard gas mixtures (Messer Group). The mixture includes H_2_ (299.1 ppm, 1,047 ppm, 18,376.3 ppm, and 2.01%), CO (103 ppm, 199.5 ppm, 994.9 ppm, and 998.6 ppm), CH_4_ (20 ppm, 198.1 ppm, 500.4 ppm, and 9,917.4 ppm), C_2_H_4_ (20 ppm, 99.4 ppm, 487.5 ppm, and 1,001.6 ppm), and C_2_H_6_ (19.9 ppm, 99.0 ppm, 521.2 ppm, and 968.2 ppm).

The liquid products were collected from the cathode and anode chambers after electrolysis and analyzed by headspace gas chromatography, high-performance liquid chromatography, and ^1^H-NMR. Headspace gas chromatography measurements were carried out using a BCHP HS-2 Headspace Sampler with GC2060 gas chromatography (Shanghai Ruimin Instrument Co., Ltd.). Typically, 10-ml vials were filled with 3 ml of the liquid sample and sealed. They were heated to 70 °C over 20 min in the headspace sampler and 1 ml of the headspace gas composition was automatically injected into the GC. The sample loop (110 °C) and transfer line (110 °C) were both heated to avoid condensation. N_2_ was used as the carrier gas. An HP-INNOWax capillary column (length: 60 m; ID: 0.32 mm; film: 0.5 μm, Agilent) was used to separate the compounds in the sample. The peak areas of methanol, ethanol, and n-propanol were converted to mole concentration (mol/L, M) using calibration curves that were obtained using methanol, ethanol, and n-propanol standard solutions (1,000 μg/ml, purchased from Beijing Yihuatongbiao Tech. Co., Ltd., and have been certified by the China National Institute of Metrology) diluted with ultra-purity water to different concentrations (0.1, 1, 5, 10, and 15 mM). In the high-performance liquid chromatography analysis (1260 Infinity II with a UV detector at 210 nm, Aglient), a mixed eluent (pH 2.5) of ultra-purity water, H_2_SO_4_ (2 mM), and Na_2_SO_4_ (25 mM) flowed through a C18-inverse column (4.6 mm × 150 mm, InertSustain AQ-C18, Shimadzu) at 1 ml/min. Before performing the analysis, the electrolyte sample is first acidified with 0.5 M H_2_SO_4_ in the same volume as the sample. The peak areas of formate and acetate were converted to mole concentration using calibration curves that were obtained using standard solutions (home-prepared) of each product at a concentration of 0.1, 2.5, 5, and 10 mM. ^1^H-NMR was performed using AVANCE IIITM HD 400 MHz NanoBAY with solvent (water) suppression. Electrolyte (400 μl) was mixed with 100 μl of a solution of 10 mM DMSO and 50 mM phenol in D_2_O as internal standards for the ^1^H-NMR analysis. The internal standards, phenol and DMSO, were chosen because they did not interfere with peaks arising from CO_2_ reduction products and because of their nonvolatility, which allowed the use and storage of the same internal standards solution for all of the product measurements without appreciable change in concentration. The area of product peaks to the right of the water peak was compared to the area of DMSO (at a chemical shift of 2.6 ppm), and the area of product peaks to the left of the water peak was compared to the area of phenol (at a chemical shift of 7.2 ppm).

### Characterization

#### Quasi in situ XPS measurements

Quasi in situ XPS studies were used to detect the chemical state of SnO*_x_*. The SnO_2_ electrodes were potentiostatically polarized in a glovebox (SUPER, MIKROUNA) using a 3-electrode PMMA cell with an IrRu alloy deposited Ti mesh counter electrode and a calibrated leak-free Ag/AgCl reference electrode. A selected potential (−1.2 V vs. RHE) was applied for a certain duration, followed by emersion under potential control and flushing with deionized water. Afterward, the specimen was transported to the XPS analyzing chamber (ESCALAB Xi+, Thermo Fisher Scientific) in a vacuum transfer module (Thermo Scientific™) for characterization of their surface chemistries, which avoids air exposure as well as surface re-oxidation. The picture of the transfer cell is shown in Fig. S17. Peak positions were further corrected by referencing the C 1s peak position of adventitious carbon for the sample (284.8 eV) and by shifting all other peaks in the spectra accordingly.

#### XRD measurements

The SnO_2_ electrodes were potentiostatically under –1.2 V vs. RHE using a 3-electrode PMMA cell with an IrRu alloy deposited Ti mesh counter electrode and a calibrated leak-free Ag/AgCl reference electrode. A selected potential was applied for different durations, followed by flushing with deionized water. The crystal structure was determined by an x-ray diffractometer (XRD, Bruker D8 Focus) with Cu Kα radiation (λ = 1.54056 Å) at 40 kV and 40 mA. XRD spectra were collected over a 2θ range of 30° to 60° at a scanning speed of 8°/min.

## Data Availability

The authors declare that in addition to the text and Supplementary Materials, the authors can provide additional supporting data.

## Supplementary Materials

Supplementary 1 Fig. S1. Three different sizes of Sn/SnO_2_(110) models. Fig. S2. (6×3) of SnO_2_(110) models and identification of 3 kinds of oxygen (O_bri_, O_ip_, and O_sub_). Fig. S3. Radial distribution functions between O and Sn atoms of Sn/SnO_2_(110). Fig. S4. Energy profile of Sn/SnO_2_(110) surface NN-MD simulation. Fig. S5. The comparison of NN potential predicted energy and that from DFT calculation of H* adsorption energy (active site: O and Sn), which means that the accuracy of NN potential can reach the level of DFT calculation. Fig. S6. Surface Pourbaix diagrams for SnO*_x_* surfaces. Fig. S7. SSW-NN global optimization of SnO_2_(110) reduction. Fig. S8. (A) Energy profile of SnO/SnO_2_(110) surface SSW-NN simulation for 5,000 steps. Fig. S9. Annealing test for Sn/SnO_2_(110). Fig. S10. Annealing test for SnO*_x_* (O_sec_). Fig. S11. Different reduced surface structures with net charge distribution, showing that as the SnO_2_(110) surface is reduced, the distribution of the net charges of Sn sites is from SnO_2_-SnO to the range of SnO-Sn. Fig. S12. The classification of the coordination number of Sn-O on SnO*_x_* surface (100%). Fig. S13. The correlation of ∆*E*_H*_, ∆*E*_COOH*_, and ∆*E*_HCOO*_ and net charge on different sites by (A) PBE and (B) PBE+D3 method. Fig. S14. *U*_L_ as a function of net charge for the 2 elementary reactions of (A) HER at Sn active sites; (B) CO_2_ER to CO at Sn active sites. Fig. S15. (A) The simulated FE of different products over SnO*_x_* surface with different reduction degrees under –1.2 V vs. RHE. Fig. S16. XRD patterns for SnO*_x_* at the reaction time of 0, 0.25, 0.5, 1.0, 1.5, and 2.0 h under –1.2 V vs. RHE. Fig. S17. The images of the transfer cell for the transport of the electrodes without exposure to air. Fig. S18. (A) Quasi in situ XPS Sn 3d spectra for SnO*_x_* at the reaction time of 0, 0.25, 0.5, 1.0, 1.5, and 2.0 h under –1.2 V vs. RHE. Fig. S19. The correlation between ∆*E*_H*_, ∆*E*_*COOH_, and ∆*E*_HCOO*_ and surface charge on model sites with different degrees of reduction. Fig. S20. The correlation between ∆*E*_H*_, ∆*E*_*COOH_, and ∆*E*_HCOO*_ and SnO_2_(101) and SnO_2_(100) surface with model sites in different degrees of reduction. Fig. S21. The correlation between integrated COHP (iCOHP) and ∆*E*_H*_, ∆*E*_*COOH_, and ∆*E*_HCOO*_ on model sites with different degrees of reduction. Fig. S22. (A to D) The projected crystal orbital Hamilton population (pCOHP) between the metal center of Sn with different degrees of reduction and O atom of HCOO* (splitting: s orbitals and s orbitals, s orbitals and p orbitals, and p orbitals and p orbitals). Fig. S23. (A to D) The projected crystal orbital Hamilton population (pCOHP) between the metal center of Sn with different degrees of reduction and O atom of HCOO*. Fig. S24. (A to D) The projected crystal orbital Hamilton population (pCOHP) between the metal center of Sn with different degrees of reduction and H atom of H* (the main splitting of interaction is 5s orbitals of Sn and 1s orbitals of H). Fig. S25. (A to D) The projected crystal orbital Hamilton population (pCOHP) between the metal center of Sn with different degrees of reduction and C atom of *COOH (the main splitting of interaction is 5s orbitals of Sn and 2p_z_ orbitals of C). Table S1. Structure information in the first-principles global dataset of the Sn-O system. Table S2. Structure information in the first-principles global dataset of the Sn-O-H system. Table S3. Comparison of adsorption energies for different functionals. Table S4. The zero-point energy correction, enthalpy correction, and entropy correction for adsorbates and free molecules. Table S5. The iCOHP of HCOO* on SnO*_x_* surfaces with different reduction degrees. Table S6. The iCOHP of *COOH on SnO*_x_* surfaces with different reduction degrees. Table S7. The iCOHP of H* on SnO*_x_* surfaces with different reduction degrees. Table S8. Periodic NBO analyses of Sn-O bonds between HCOO* and tin oxide surfaces. Table S9. Periodic NBO analyses of Sn-C bonds between *COOH and tin oxide surfaces. References [49,50]
