# Supplementary material for: Neural Network Accelerated Investigation of the Dynamic Structure–Performance Relations of Electrochemical CO2 Reduction over SnOx Surfaces
Source: Research (Wash D C). 2023 Mar 14;6:0067. doi: 10.34133/research.0067 (PMC10013797; doi:10.34133/research.0067)
Supplement: Supplementary 1 — Fig. S1. Three different sizes of Sn/SnO2(110) models. Fig. S2. (6×3) of SnO2(110) models and identification of 3 kinds of oxygen (Obri, Oip, and Osub). Fig. S3. Radial distribution functions between O and Sn atoms of Sn/SnO2(110). Fig. S4. Energy profile of Sn/SnO2(110) surface NN-MD simulation. Fig. S5. The comparison of NN potential predicted energy and that from DFT calculation of H* adsorption energy (active site: O and Sn), which means that the accuracy of NN potential can reach the level of DFT calculation. Fig. S6. Surface Pourbaix diagrams for SnOx surfaces. Fig. S7. SSW-NN global optimization of SnO2(110) reduction. Fig. S8. (A) Energy profile of SnO/SnO2(110) surface SSW-NN simulation for 5,000 steps. Fig. S9. Annealing test for Sn/SnO2(110). Fig. S10. Annealing test for SnOx (Osec). Fig. S11. Different reduced surface structures with net charge distribution, showing that as the SnO2(110) surface is reduced, the distribution of the net charges of Sn sites is from SnO2-SnO to the range of SnO-Sn. Fig. S12. The classification of the coordination number of Sn-O on SnOx surface (100%). Fig. S13. The correlation of ∆EH*, ∆ECOOH*, and ∆EHCOO* and net charge on different sites by (A) PBE and (B) PBE+D3 method. Fig. S14. UL as a function of net charge for the 2 elementary reactions of (A) HER at Sn active sites; (B) CO2ER to CO at Sn active sites. Fig. S15. (A) The simulated FE of different products over SnOx surface with different reduction degrees under –1.2 V vs. RHE. Fig. S16. XRD patterns for SnOx at the reaction time of 0, 0.25, 0.5, 1.0, 1.5, and 2.0 h under –1.2 V vs. RHE. Fig. S17. The images of the transfer cell for the transport of the electrodes without exposure to air. Fig. S18. (A) Quasi in situ XPS Sn 3d spectra for SnOx at the reaction time of 0, 0.25, 0.5, 1.0, 1.5, and 2.0 h under –1.2 V vs. RHE. Fig. S19. The correlation between ∆EH*, ∆E*COOH, and ∆EHCOO* and surface charge on model sites with different degrees of reduction. Fig. S20. The corre [file research.0067.f1.pdf]

Supporting Information

Neural Network Accelerated Investigation of the Dynamic Structure-Performance Relations of Electrochemical CO_2 Reduction over SnO_x Surfaces

Lulu Li[1,2,3,4#], Zhi-Jian Zhao[1,2,3,4#], Gong Zhang[1,2,3,4], Dongfang Cheng[1,2,3,4], Xin Chang[1,2,3,4], Xintong Yuan[1,2,3,4], Tuo Wang[1,2,3,4,5], and Jinlong Gong[1,2,3,4]*

[1]*School of Chemical Engineering and Technology, Key Laboratory for Green Chemical Technology of Ministry of Education, Tianjin University, Tianjin 300072, China.*

[2]*Collaborative Innovation Center for Chemical Science & Engineering (Tianjin), Tianjin 300072, China.*

[3]*National Industry-Education Platform of Energy Storage, Tianjin University, Tianjin 300072, China.*

[4]*Haihe Laboratory of Sustainable Chemical Transformations, Tianjin 300192, China.*

[5]*Joint School of National University of Singapore and Tianjin University, International Campus of Tianjin University, Binhai New City, Fuzhou 350207, China.*

*Email: jlgong@tju.edu.cn

Table of Contents

1. Material and Methods

2. Supplementary Figures and Tables

3. References

Materials and Methods

Computational Details of SSW-NN and MD-NN simulations

As a means to determine the model size for the simulation of SnO_2 reduction process by SSW-NN and MD-NN method, three different models are constructed initially (namely, 4×2, 6×3, 8×4 surfaces). (6×3) of $SnO_2(110)$ surface, which is the middle size model, is considered as suitable model to detect the reduction process (Figure S1). Since $SnO_2(110)$ has three oxygen species, bridging oxygen (O_{bri}), in-plane oxygen (O_{ip}), and first layer subsurface oxygen (O_{sub}), where O_{bri} has been considered to be thermodynamically stable when present in the hydroxyl ($O_{bri}H$) form, and O_{ip} is the oxygen vacancy (O_{v_ip}) form.[1] Therefore, 0% in the reduction of $SnO_2(110)$ is the surface at the $O_{bri}H$-termination, and the presence of OH is no longer considered for the other reduction degree.

Computational Details of DFT calculations

It worth noticing that PBE functional may give misleading results for CO and CO_2 interactions with metal/metal oxide surfaces. In order to clarify the effect of dispersion corrected functionals on adsorption energies, we compared the adsorption energies by PBE and PBE+D3, and the results are shown in Table S3. The adsorption energy difference is within the DFT error. Moreover, the correlation between net charge and adsorption energy (calculated by PBE+D3) is also consistent with the previous linear relationship, with a constant slope and a change in the intercept with the correction of D3, which implies that the trend of adsorption energy variation is unaffected (see Figure S12). Therefore, the results obtained by PBE method are acceptable.

The surface Pourbaix diagram is calculated in this work by the following process:

$SnO_x + 2nH^+ + 2ne^- \rightarrow SnO_{x-n} + nH_2O$;

Besides, $\Delta G_{reaction} = nGH_2O + ESnO_{x-n} - nGH_2 - ESnO_x$. If the pH effect is considered, the

$\Delta G_{reaction}$ should also include pH correction term, that is, -ln(10)*K_B*T*pH.[2] Actually, the pH correction will only make the overall vertical translation of the surface Pourbaix diagram, and will not cause changes in the whole trend (see Figure S6).

In this work, we consider both electrochemical CO_2 reduction (CO_2ER) and hydrogen evolution reaction (HER). The asterisk (*) denotes the substrate. The whole process including the CO_2ER to formate or CO and the competing HER to H_2 consists of the following main steps:

For CO_2ER to formate, the first step is CO_2 (g) + * + H^+ (aq) + e^- → $HCOO^*$, ΔG_{R1} = G_{HCOO*} – 1/2G_{H2} – G_{CO2}, which is also the formula of the adsorption free energy of HCOO*(ΔG_{HCOO*}), the second step is $HCOO^*$ + H^+ (aq) + e^- → HCOOH (aq), ΔG_{R2} = G_{HCOOH} (aq) – 1/2G_{H2} – G_{HCOO*}, that is, $G_{equilibrium(HCOOH)}$ – ΔG_{HCOO*}.

The same goes for CO_2ER to CO. The first step is CO_2 (g) + * + H^+ (aq) + e^- → $COOH^*$, ΔG_{R3} = G_{*COOH} – 1/2G_{H2} – G_{CO2}, which also means ΔG_{COOH*}, the second step is *COOH + H^+ (aq) + e^- → CO (g) + H_2O (l), ΔG_{R4} = G_{CO} +G_{H2O} – 1/2G_{H2} – G_{COOH*}, considered directly as $G_{equilibrium(CO)}$ – ΔG_{COOH*}.

For HER, ΔG_{H*} is considered as the major descriptor of HER, and |ΔG_{H*}| is numerically equal to the U_L of HER.

Therefore, the largest Gibbs free energy change (ΔG_{max}) of CO_2RR to formate is max{ΔG_{R1}, ΔG_{R2}}, of CO_2RR to CO is max{ΔG_{R3}, ΔG_{R4}} and of HER is |ΔG_{R5}|.

Theoretical current density and faradaic efficiency calculation

The theoretical current densities of CO_2ER and HER can be simulated by the following formulations[3]:

$$J^{HCOOH}(U) = \frac{1}{N_{surf}} J_0^{HCOOH} \sum_{i=1}^{N_{surf}} \exp\left[-\frac{1}{k_B T}\Delta G_i^{HCOOH}\right] \quad (1\text{-}1)$$

$$J^{CO}(U) = \frac{1}{N_{surf}} J_0^{CO} \sum_{i=1}^{N_{surf}} \exp\left[-\frac{1}{k_B T}\Delta G_i^{CO}\right] \quad (1\text{-}2)$$

$$J^{H2}(U) = \frac{1}{N_{surf}} J_0^{H2} \sum_{i=1}^{N_{surf}} \exp\left[-\frac{1}{k_B T}\Delta G_i^{H2}\right] \quad (1\text{-}3)$$

Among them, U is the external bias potential which is defined versus the reversible hydrogen electrode (RHE), N_{surf} is the number of surface atoms exposed to the different reduced degree surfaces. k_B is the Boltzmann constant and T is the reaction temperature. Besides, $\Delta G_i^{formate}$, ΔG_i^{CO}, and ΔG_i^{H2} mean the highest thermodynamic energy barriers of the CO_2ER to formate and CO as well as HER. It is worth noting that we use the energy of the potential-limiting step to represent $\Delta G_i^{formate}$, ΔG_i^{CO}, and ΔG_i^{H2} in this work. $J_0^{formate}$, J_0^{CO}, and J_0^{H2} are described for the prefactors of CO_2ER to formate and CO as well as HER, which can be obtained from the experiment at -1.2 V vs RHE.[4] This leads to $J_0^{formate} = 7.63 \times 10^{-6}$ mA/cm^{-2}, $J_0^{CO} = 3.31 \times 10^{-11}$ mA·cm^{-2}, and $J_0^{H2} = 1.28 \times 10^{-14}$ mA/cm^{-2}. As for the theoretical Faradaic efficiency, it can be obtained by the following forms:

$$FE_{HCOOH} = \frac{J^{HCOOH}(U)}{J^{HCOOH}(U)+J^{CO}(U)+J^{H2}(U)} \quad (1\text{-}4)$$

$$FE_{CO} = \frac{J^{CO}(U)}{J^{HCOOH}(U)+J^{CO}(U)+J^{H2}(U)} \quad (1\text{-}5)$$

$$FE_{H2} = \frac{J^{H2}(U)}{J^{HCOOH}(U)+J^{CO}(U)+J^{H2}(U)} \quad (1\text{-}6)$$

We counted the number of surface atoms (N_{surf}) exposed to the different reduced degree surfaces and corresponding net charge five times every 200 fs during the last 0.1 ns MD-NN simulation, which stage has already reached dynamic equilibrium. Then through the linear relationship between the net charge and adsorption energy, the adsorption energy of key intermediates can be simulated directly, without any DFT calculation. Combined with free energy correction, solvation effect correction, the theoretical limiting potentials of CO_2ER to

formate, CO and HER at different active sites are obtained, which can be used directly to simulate the average theoretical FE as we mentioned above.

Experimental Materials

IrRu alloy deposited Ti mesh (200 mesh) was purchased from Xian Taijin Industrial Electrochemical Technology Co.,LTD. Commercially available carbon-based gas diffusion layers (GDLs, AvCarb GDS3250) were purchased from Xima Laya Photo-Electric Technology Co., Ltd., China. $KHCO_3$ (≥99.99%, metal basis), H_2SO_4 (99.999%, metal basis), Na_2SO_4 (99.999%, metal basis), CH_3OH (≥99.9%, LC-MS), dimethyl sulfoxide (DMSO, ≥ 99.95%, standard for GC), D_2O (100%, 99.96 atom % D), isopropyl alcohol (99.999%, trace metals basis), HCOONa (99.998%, metal basis), CH_3COONa (99.99%, metal basis), SnO_2 (99.99%, metal basis, 50-70 nm) were purchased from Sigma-Aladdin. The reagents were used without any purification process. Ultra-purity water (18.25 MΩ·cm) supplied by a Millipore Direct-Q5 System was used in the whole experimental process. CO_2 was supplied by Air Liquide (≥99.999%).

Supplementary Figures and Tables

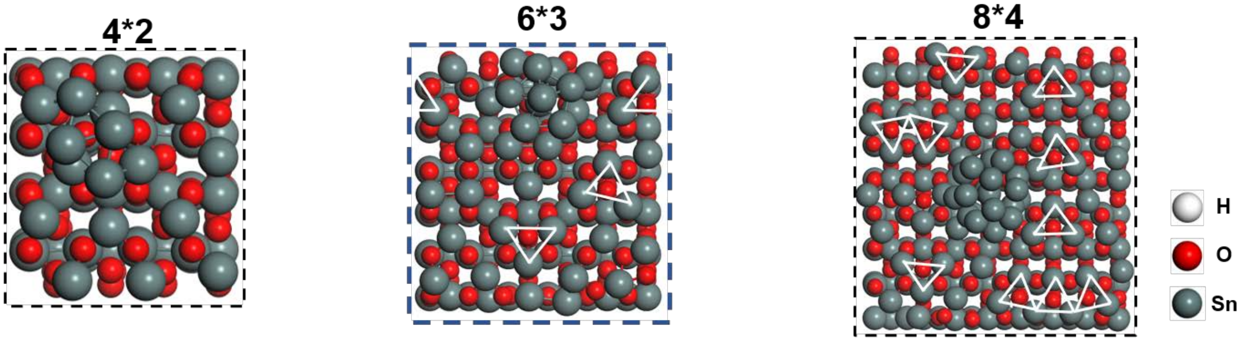

Figure S1. Three different sizes of $Sn/SnO_2(110)$ models.

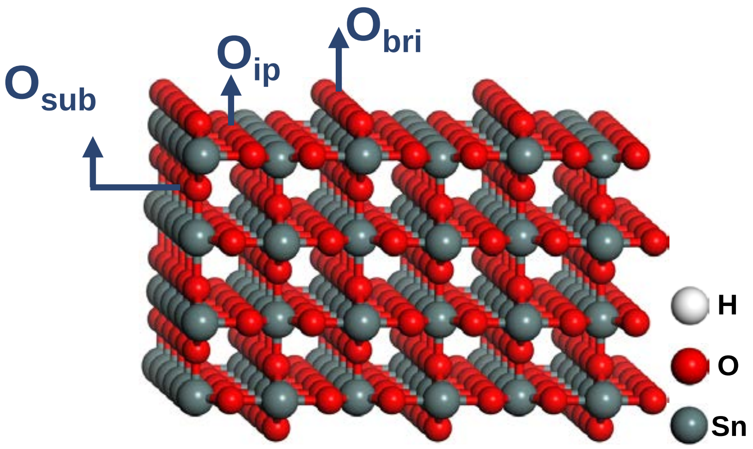

Figure S2. (6×3) of SnO_2(110) models and identification of three kinds of oxygen (O_{bri}, O_{ip}, and O_{sub}).

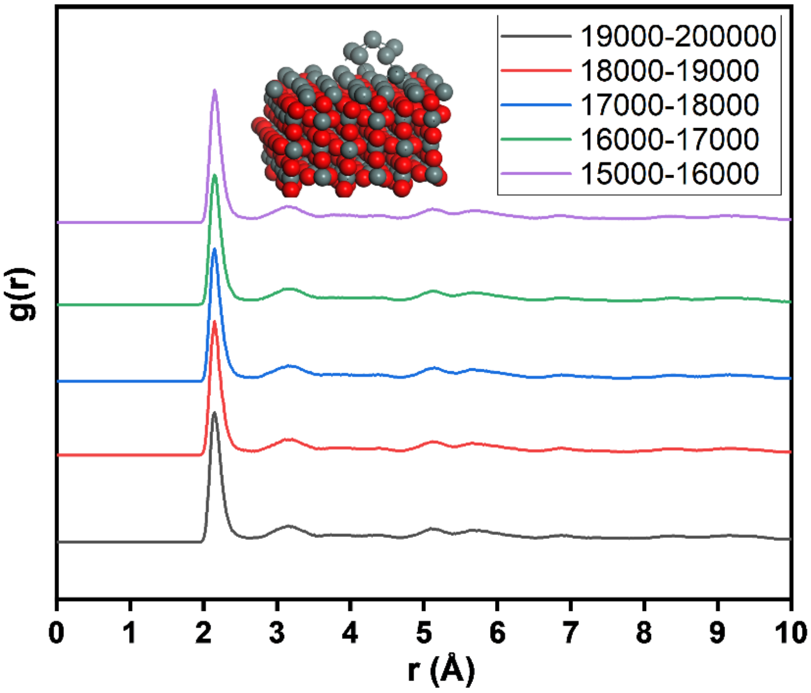

Figure S3. Radius distribution functions (RDFs) between O and Sn atoms of Sn/SnO_2(110).

We selected last 0.5 ns and every 0.1 ns are used to perform RDFs to compare the equilibrium. It can be found that the trends of five times RFDs are the same, which means the system are in equilibrium and 2 ns is enough to simulate the MD-NN.

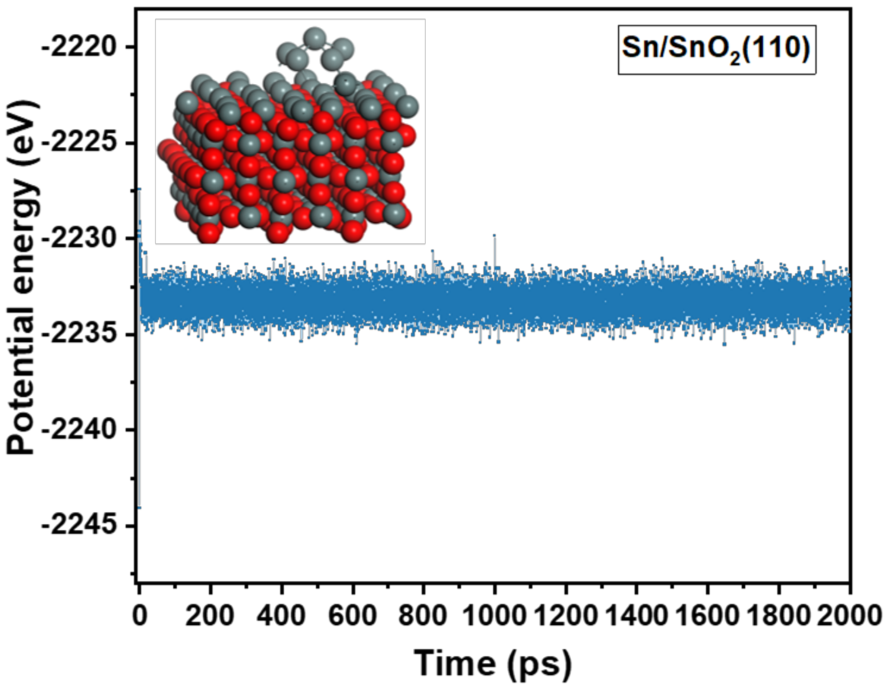

Figure S4. Energy profile of $Sn/SnO_2(110)$ surface NN-MD simulation.

The insert shows the structure of $Sn/SnO_2(110)$ after 2 ns of NN-MD simulation. It can be clearly indicated that the point of potential energy starts to oscillate around a constant quickly after the beginning of the MD simulation and the time scale of 2 ns can guarantee the energy equilibrium of the MD simulation.

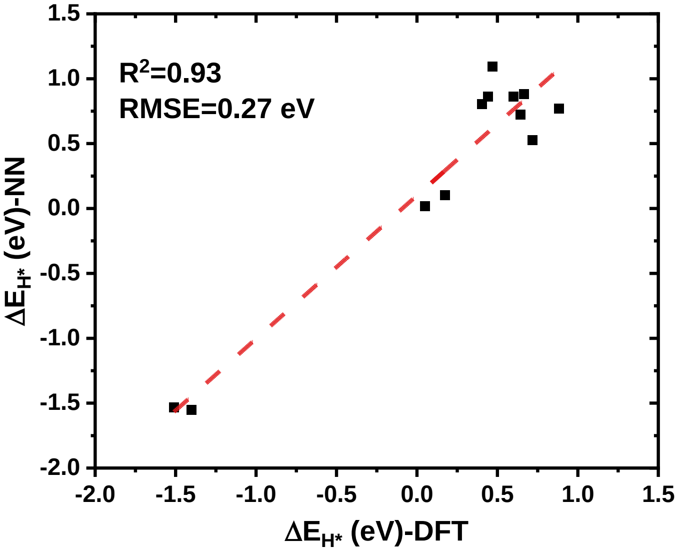

Figure S5. The comparison of NN potential predicted energy and that from DFT calculation of H* adsorption energy (active site: O and Sn), which means that the accuracy of NN potential can reach the level of DFT calculation[5].

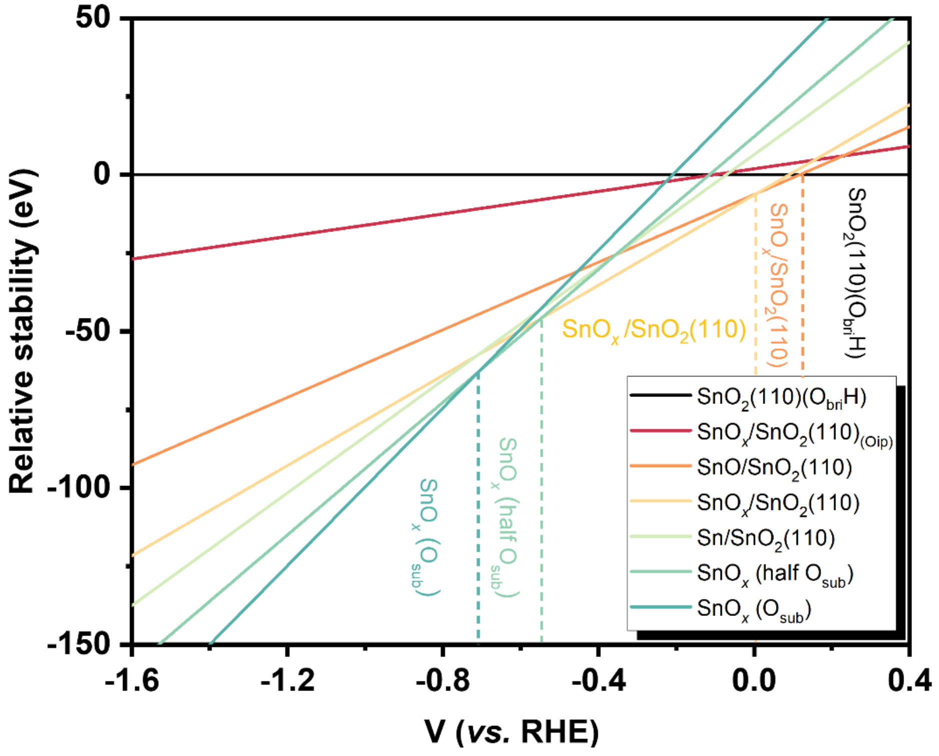

Figure S6. Surface Pourbaix diagrams for SnO_x surfaces. SnO_2(110) (O_{bri}H), SnO_x/SnO_2(110), SnO_x/SnO_2(110), SnO_x(half O_{sub}) and SnO_x(O_{sub}) can be stable under different potentials. Besides, this trend is valid under different pH conditions.

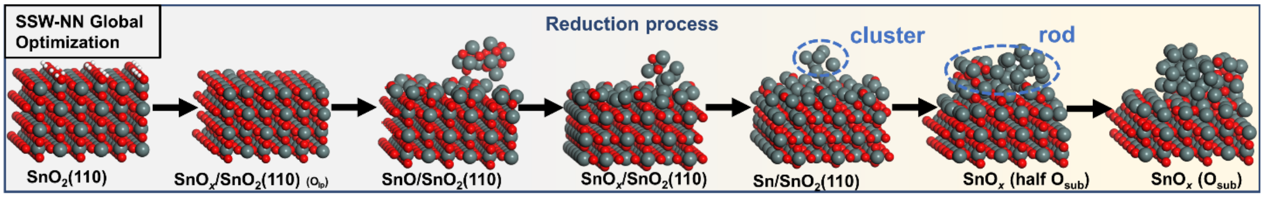

Figure S7. SSW-NN global optimization of $SnO_2(110)$ reduction.

The trends are similar during the reduction process by the SSW-NN method. Some differences such as O evolution and aggregation with Sn atoms occur with SSW-NN optimization, indicating the diffusion barrier of sub-surface O to surface might sluggish the reduction of SnO_2 during the MD-NN simulation.

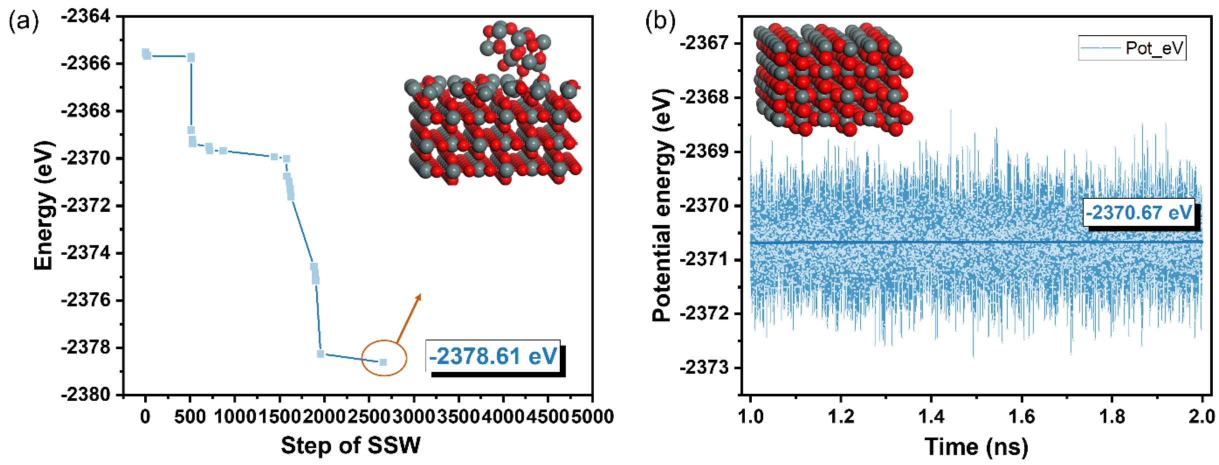

Figure S8. (a) Energy profile of $SnO/SnO_2(110)$ SSW-NN simulation for 5000 steps. (b) Energy profile of $SnO/SnO_2(110)$ MD-NN simulation in 1-2 ns.

In Figure S8(a), it can be found that the global minima are determined at step 2658 and are similar to the energy at step 1967. Moreover, the configuration obtained by the optimization of 5000 steps is similar to the $SnO/SnO_2(110)$ structure in Figure S7. A comparison with the average potential energy of MD-NN in Figure S8(b) shows that the energy of the configuration obtained using global optimization is about 8 eV lower than that of the MD-NN configuration, demonstrating that the SSW-MD method is more focused on searching for thermodynamically stable configurations.

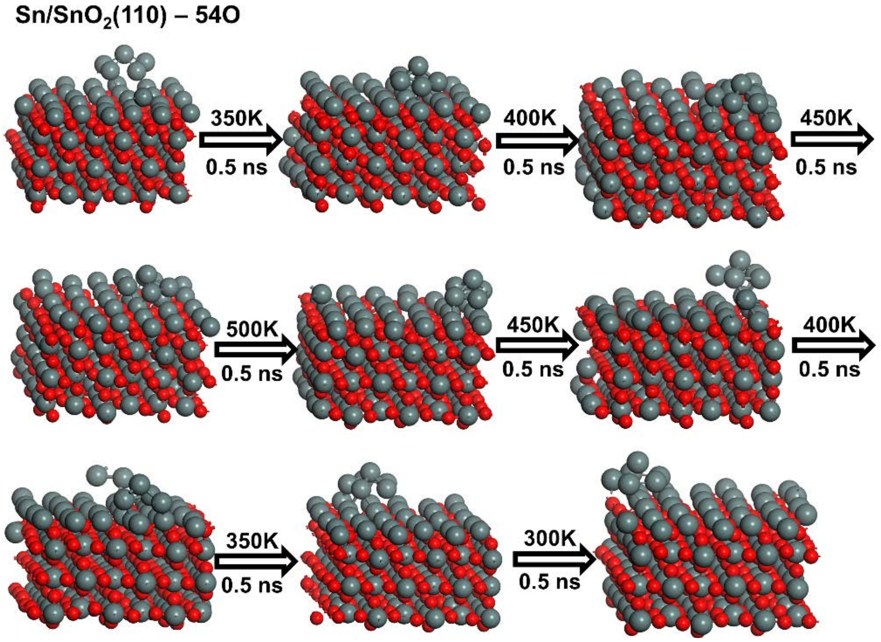

Figure S9. Annealing test for $Sn/SnO_2(110)$. The annealing temperature increased from 300 K to 500 K and then cooled down to 300 K.

In the annealing process, the temperature was increased every 50 K, and the MD-NN is used to optimize the structure for 0.5 ns each time in vacuum. The annealing test did not lead to significant reconstruction of $Sn/SnO_2(110)$ (75%), indicating that the structure obtained from the simulations is kinetically and thermodynamically stable.

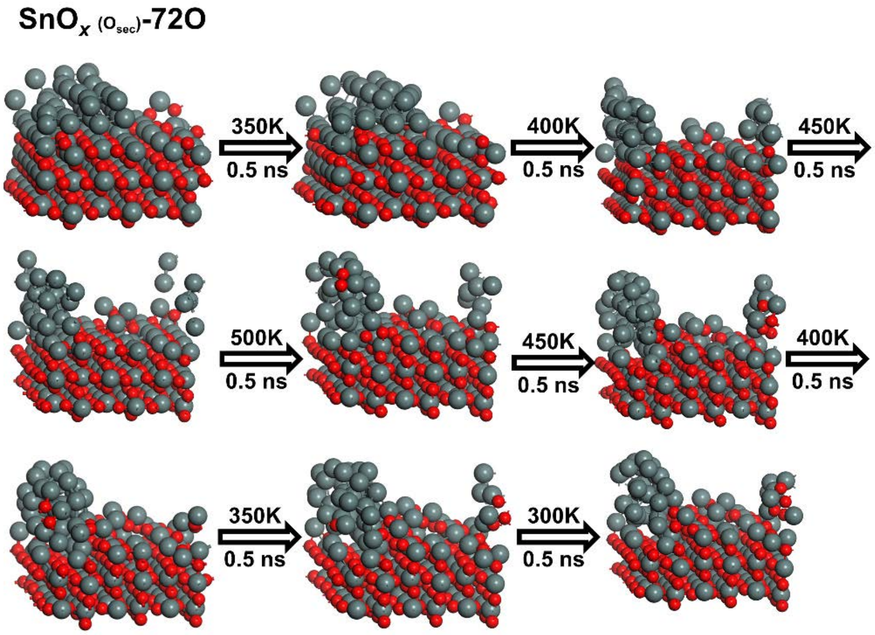

Figure S10. Annealing test for SnO_x (O_{sec}). The annealing temperature increased from 300 K to 500 K and then cooled down to 300 K.

In the annealing process, the temperature was increased every 50 K, and the MD-NN is used to optimize the structure for 0.5 ns each time in vacuum. The results show that as the reduction occurred, annealing can be used to conquer the energy barrier of MD-NN simulation and have a tendency to form a similar structure with SSW-NN. The results show that as the reduction occurred, annealing can be used to conquer the energy barrier of MD-NN simulation and have a tendency to form a similar structure with SSW-NN.

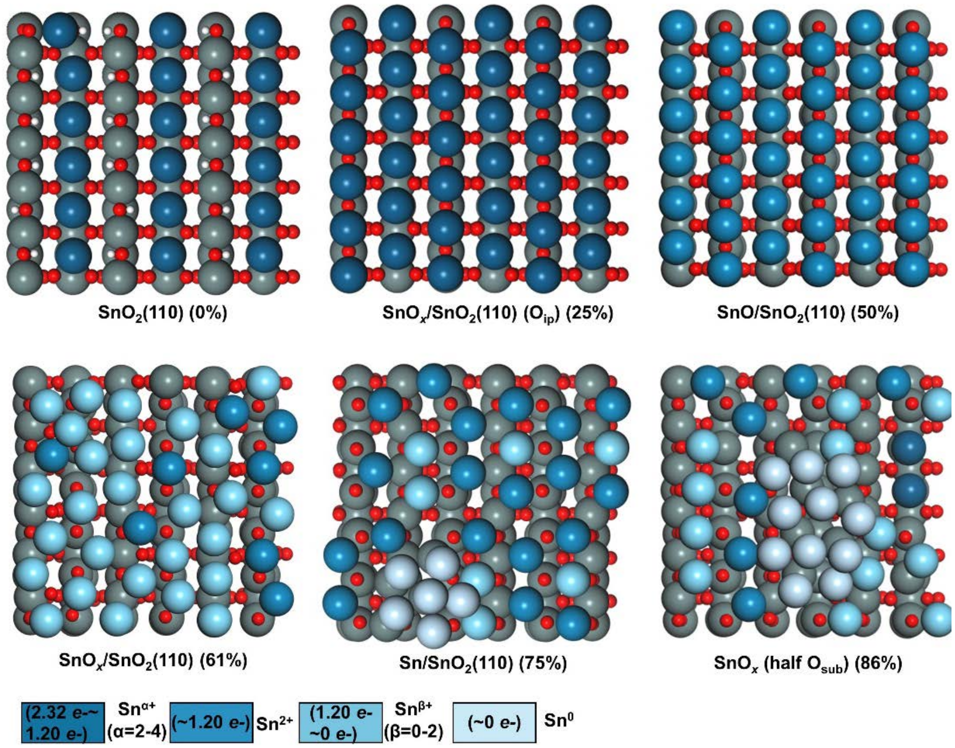

Figure S11. Different reduced surface structures with net charge distribution showing that as the SnO_2(110) surface is reduced the distribution of the net charges of Sn sites is from SnO_2-SnO to the range of SnO-Sn.

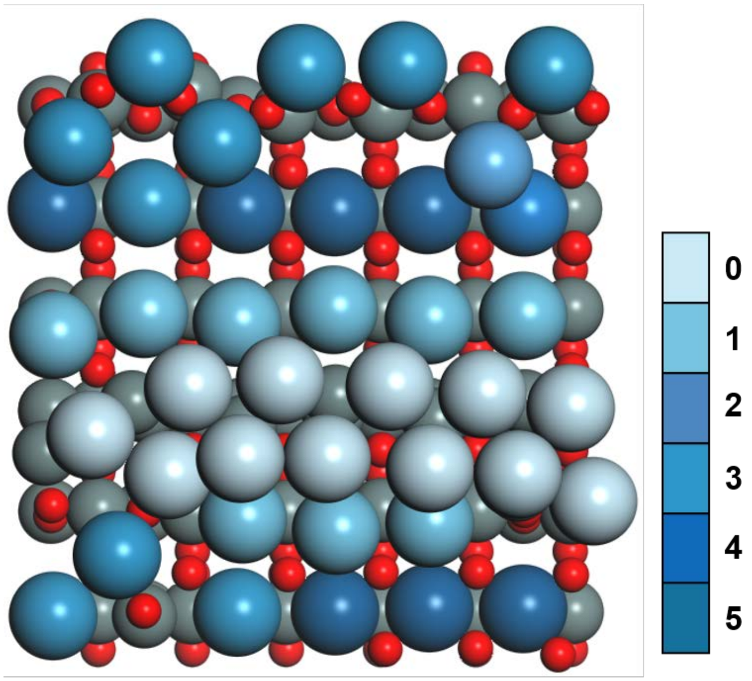

Figure S12. The classification of the coordination number of Sn-O on SnO_x surface (100%).

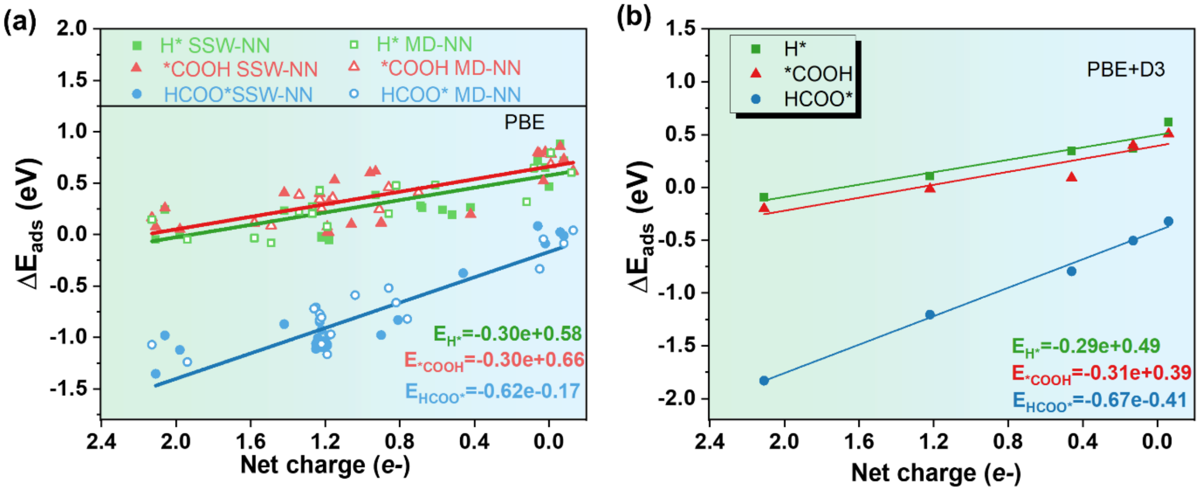

Figure S13. The correlation of ΔE_{H^*}, ΔE_{COOH^*}, and ΔE_{HCOO^*} and net charge on different sites by (a) PBE and (b) PBE+D3 method.

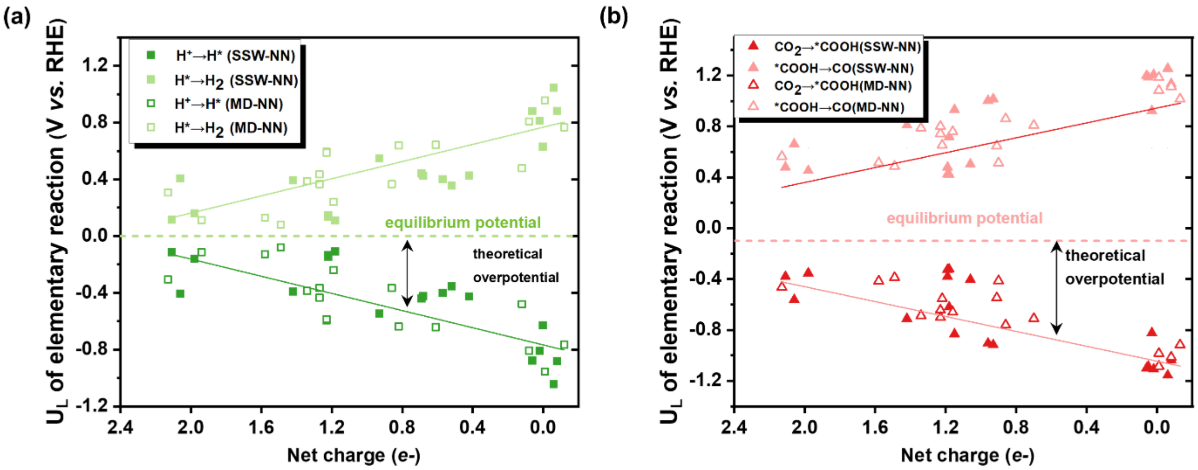

Figure S14. U_L as a function of net charge for the two elementary reactions of (a) HER at Sn active sites; (b) CO_2ER to CO at Sn active sites.

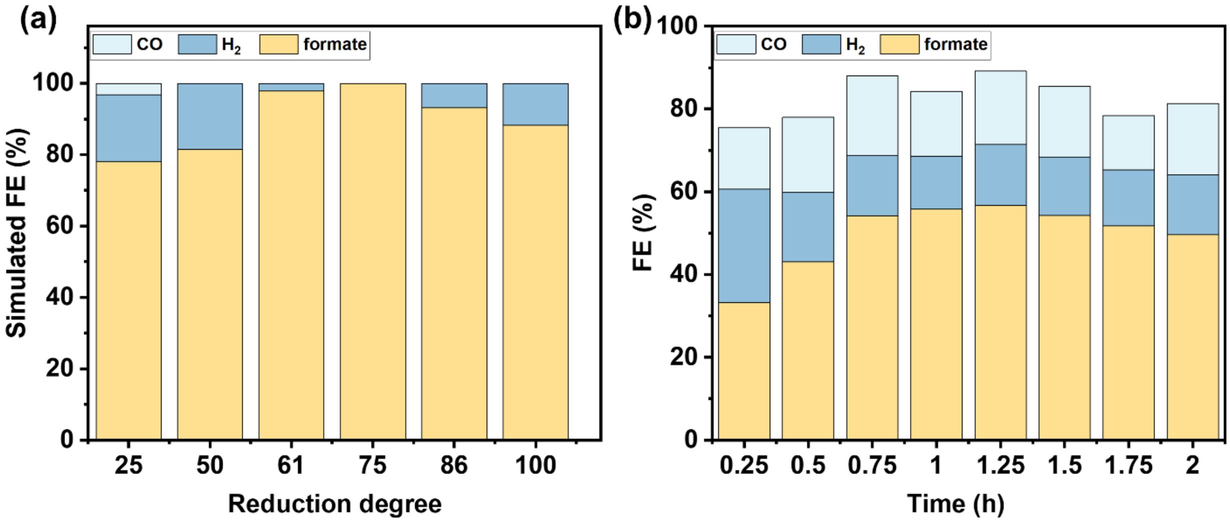

Figure S15. (a) The simulated FE of different products over SnO_x surface with different reduction degree under –1.2 V vs RHE. (b) FE of different products over SnO_x during the reaction duration under –1.2 V vs RHE.

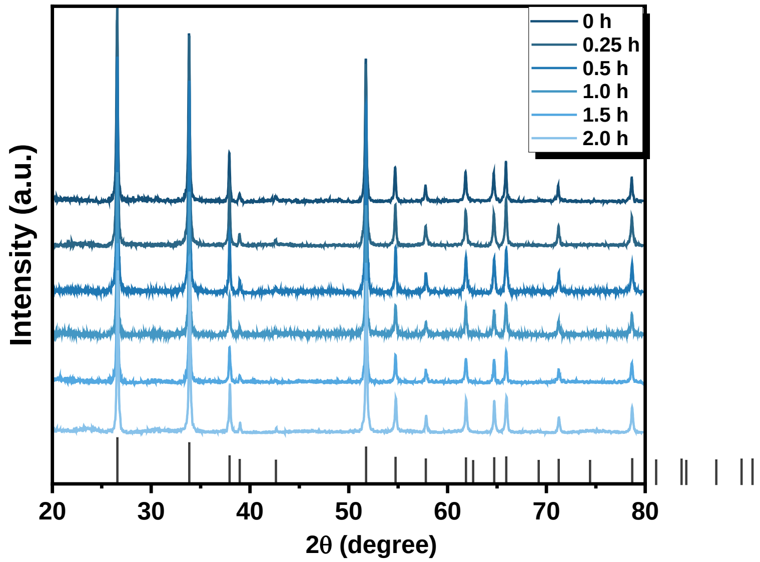

Figure S16. XRD patterns for SnO_x at the reaction time of 0, 0.25, 0.5, 1.0, 1.5, 2.0 hours under –1.2 V vs RHE.

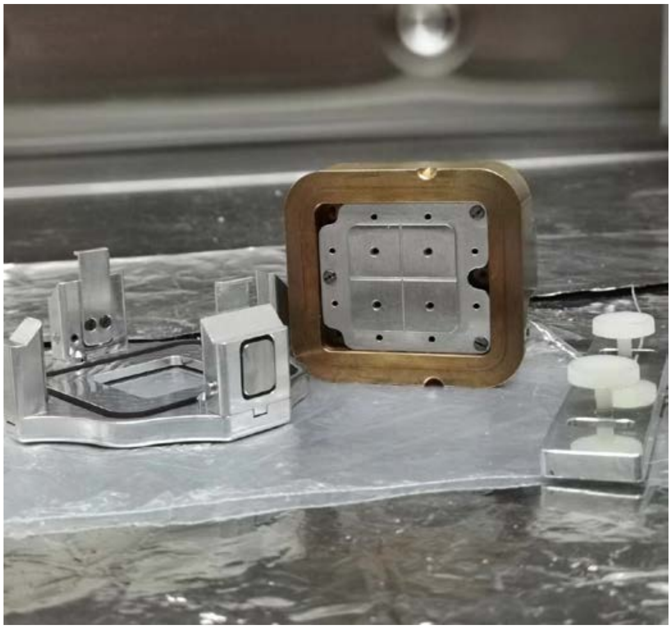

Figure S17. The images of the transfer cell for the transport of the electrodes without exposure to air.

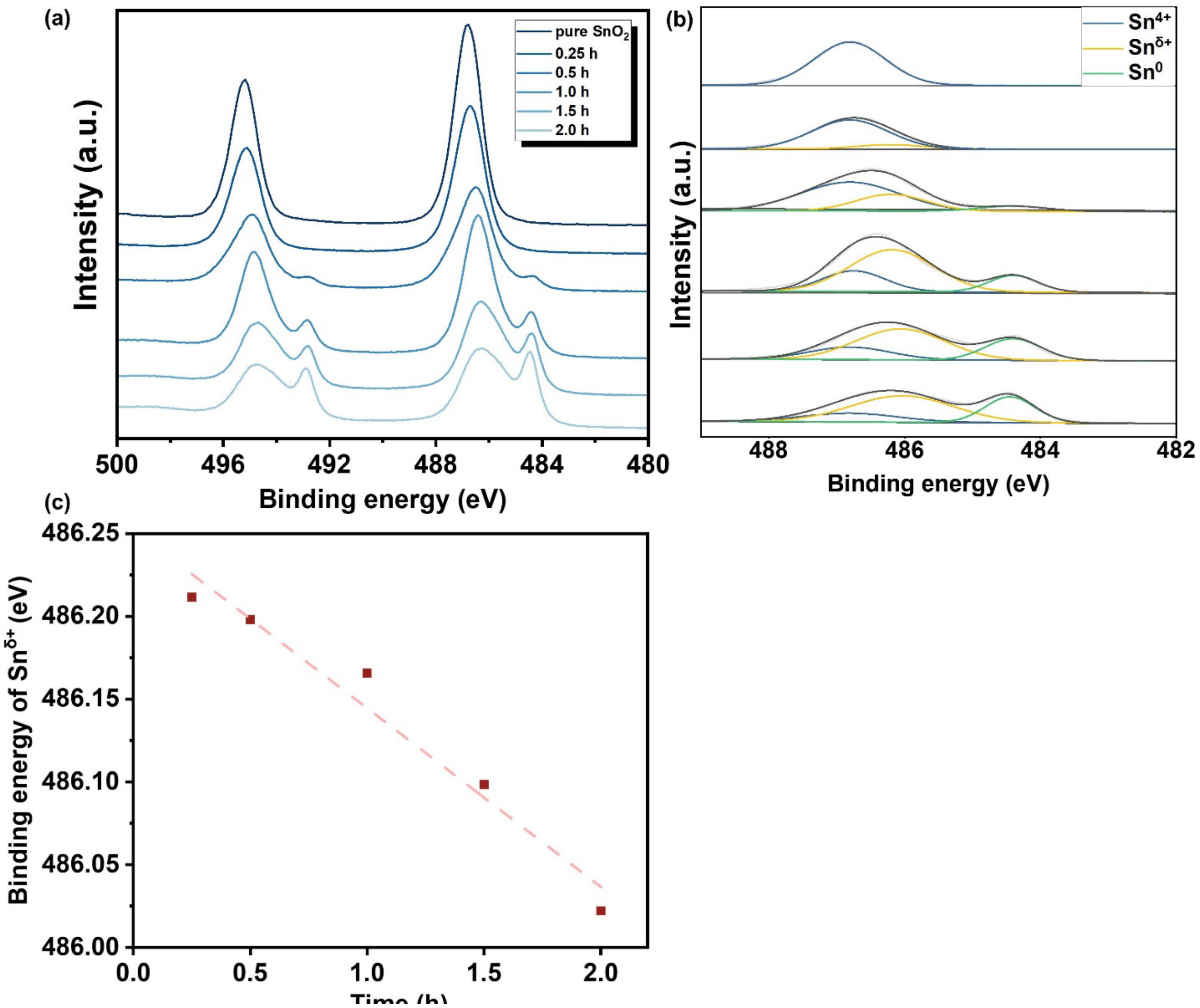

Figure S18. (a) *Quasi in-situ* XPS Sn 3d spectra for SnO_x at the reaction time of 0, 0.25, 0.5, 1.0, 1.5, 2.0 hours under –1.2 V vs RHE. (b) The XPS spectra of Sn $3d_{5/2}$ region of SnO_x, Sn^{4+}: 486.8 eV and Sn^0: 484.4 eV.[6, 7] Since the binding energy $Sn^{\delta+}$ is ambiguous, the position is not fixed, but determined by the overall fit. (c) The correlation of the reaction time with the binding energy of $Sn^{\delta+}$, which means the reduction of SnO_x as reaction time increases.

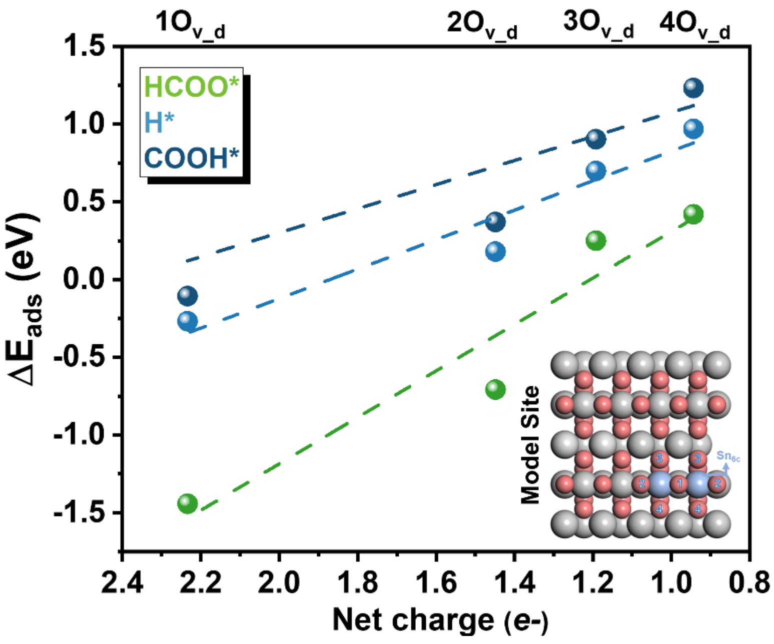

Figure S19. The correlation between ΔE_{H*}, ΔE_{*COOH}, and ΔE_{HCOO*} and surface charge on model sites with different degrees of reduction.

ΔE_{ads} decreases with the increasement of the net charge, in line with the above relationship (inset is the model active sites constructed by removing the oxygen atoms from the symmetrical positions of the two Sn_{6c} atoms due to the bidentate adsorption formation of HCOO*).

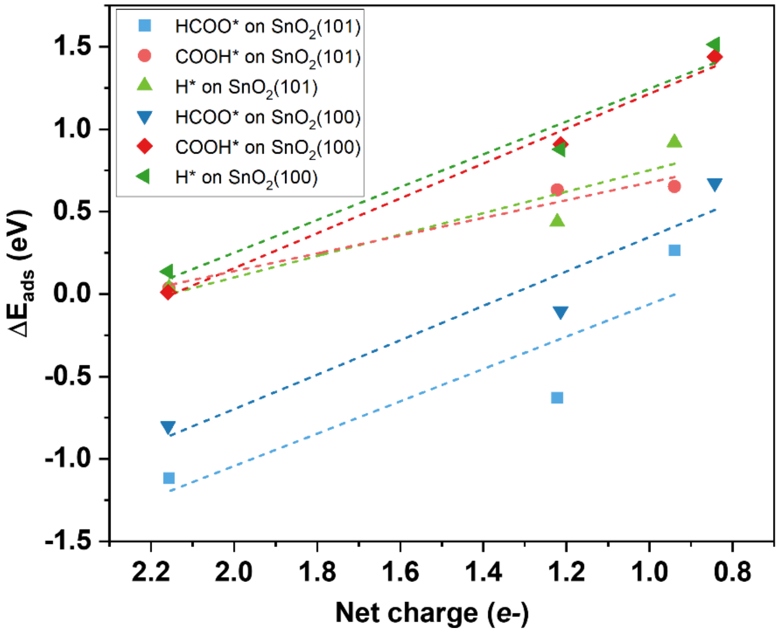

Figure S20. The correlation between ΔE_{H*}, ΔE_{*COOH}, and ΔE_{HCOO*} and SnO_2(101) and SnO_2(100) surface with model sites in different degrees of reduction.

ΔE_{ads} decreases with the increasing net charge, in line with the above relationship. The slope is different with SnO_2(110), which may be due to the structural differences on different facets since the calculation of SnO_2(101) and SnO_2(100) only uses local structural optimization and without the consideration of surface reconstruction.

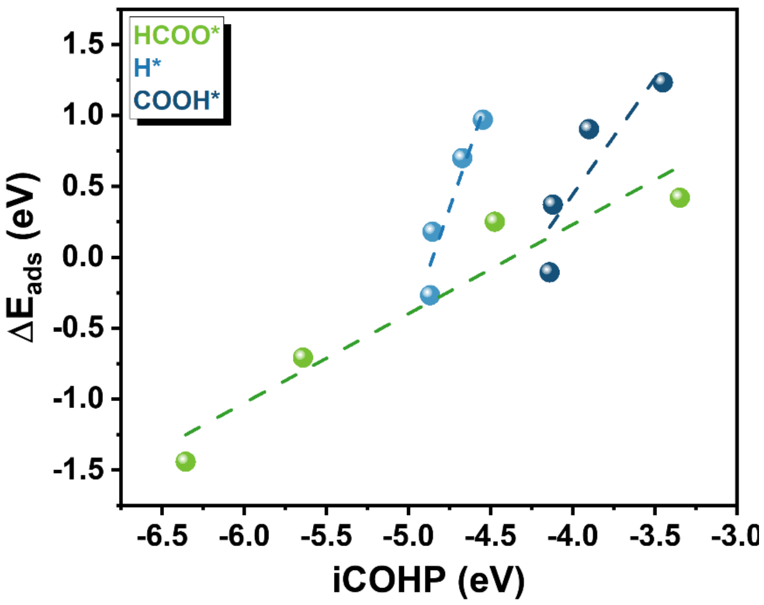

Figure S21. The correlation between integrated COHP (iCOHP) and ΔE_{H*}, ΔE_{*COOH}, and ΔE_{HCOO*} on model sites with different degrees of reduction.

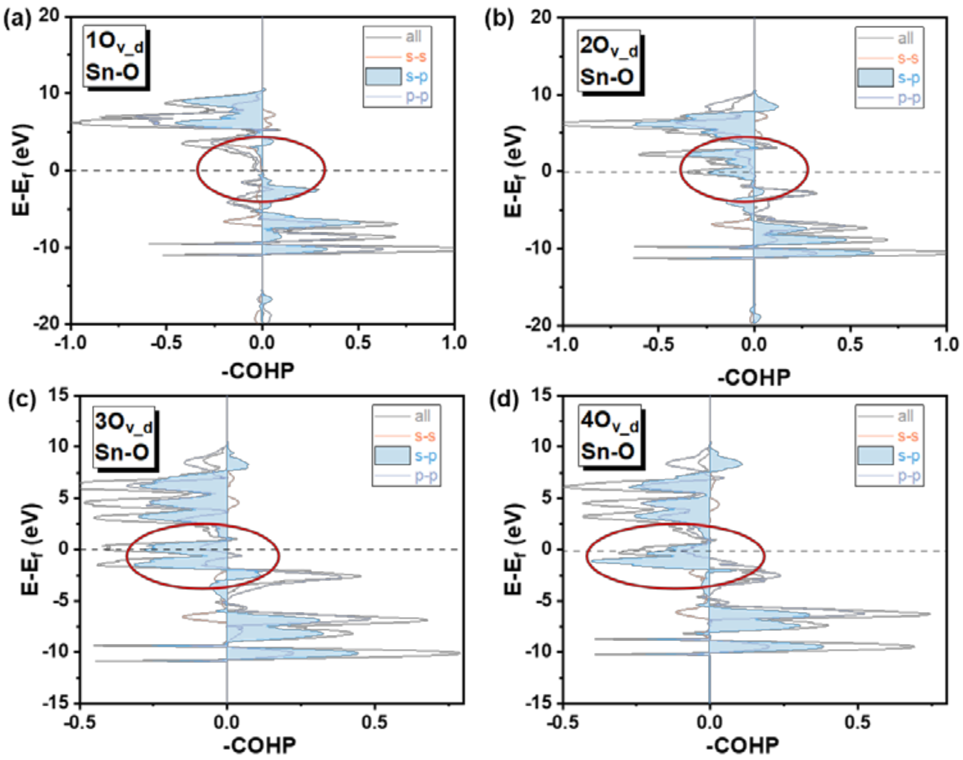

Figure S22. (a)-(d) The projected crystal orbital Hamilton population (pCOHP) between the metal center of Sn with different degrees of reduction and O atom of HCOO* (splitting: s orbitals and s orbitals, s orbitals and p orbitals, p orbitals and p orbitals).

The projected COHP (pCOHP) was further performed to explain the change of iCOHP by localizing into the specific orbital populations. We first decomposed the COHP into s-s orbitals, s-p orbitals, and p-p orbitals of Sn and O atoms. Clearly, the interaction of s-p orbitals has the dominant occupation, in other words, s-p orbitals play a major role in the whole Sn-O bonding. These results preliminarily illustrate the filling degree of the s-p anti-bonding orbital increases was the reason that weaker interaction of Sn-O bonding occurs when the O_v increases.

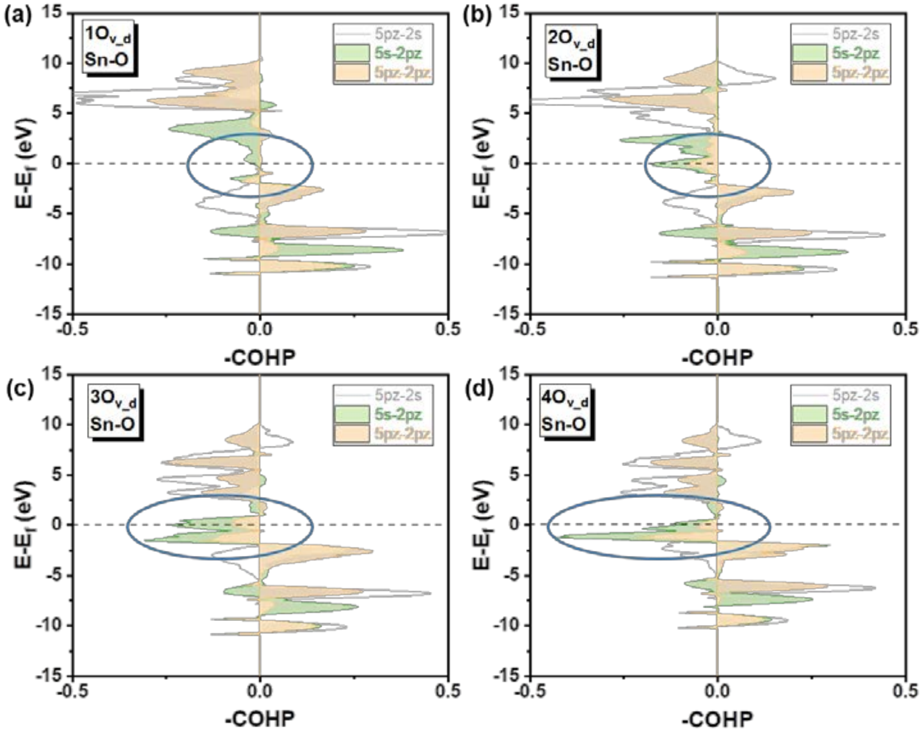

Figure S23. (a)-(d) The projected crystal orbital Hamilton population (pCOHP) between the metal center of Sn with different degrees of reduction and O atom of HCOO*.

The main splitting of interaction between s orbitals and p orbitals from Figure S21, which are $5p_z$ orbitals of Sn and 2s orbitals of O, 5s orbitals of Sn and $2p_z$ orbitals of O, $5p_z$ orbitals of Sn and $2p_z$ of O. The anti-bonding states near and below the Fermi level are mainly composed of Sn 5s as well as Sn $5p_z$, interacted with O $2p_z$ orbitals.

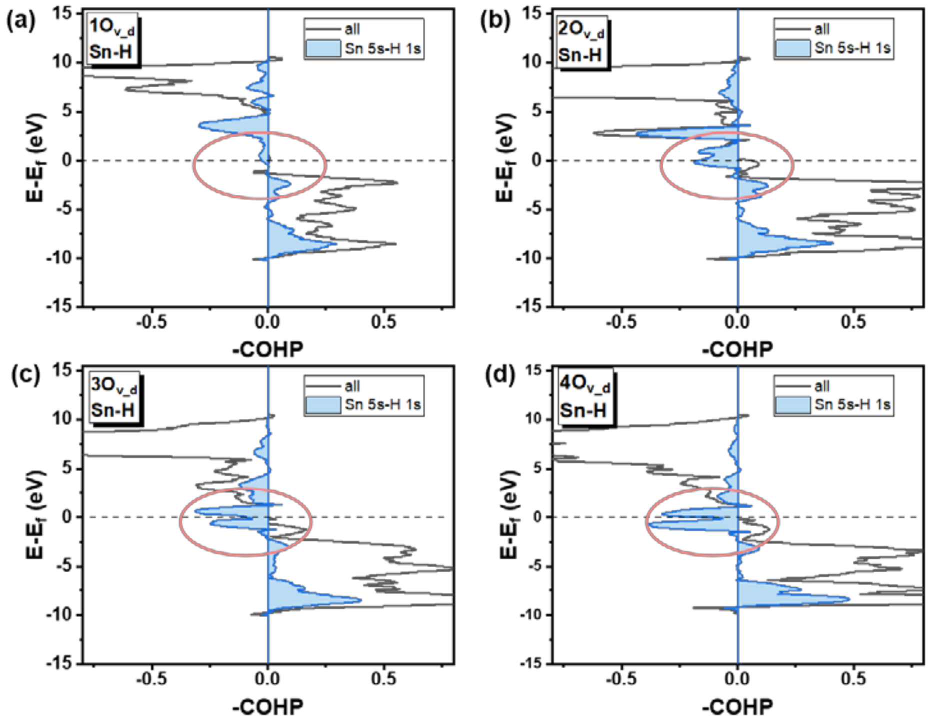

Figure S24. (a)-(d) The projected crystal orbital Hamilton population (pCOHP) between the metal center of Sn with different degrees of reduction and H atom of H* (the main splitting of interaction is 5s orbitals of Sn and 1s orbitals of H).

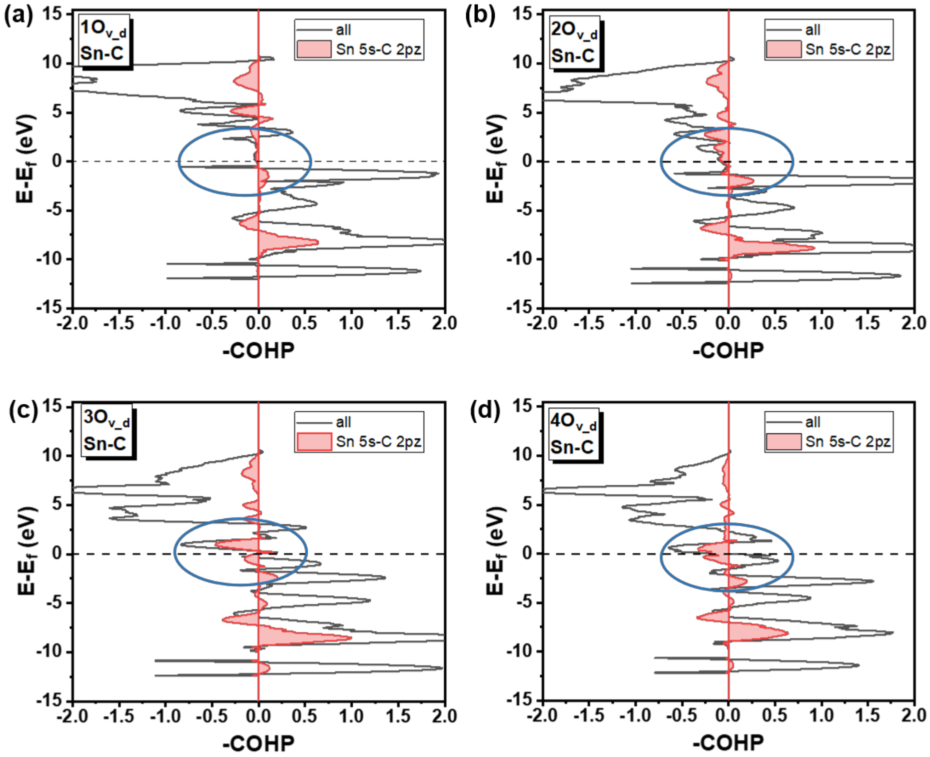

Figure S25. (a)-(d) The projected crystal orbital Hamilton population (pCOHP) between the metal center of Sn with different degrees of reduction and C atom of *COOH (the main splitting of interaction is 5s orbitals of Sn and $2p_z$ orbitals of C).

Table S1. Structure information in the first principles global dataset of Sn-O system. Listed data are the number of the structures in the global dataset, as distinguished by the chemical formula, the number of atoms per cell (Natom), the type of structures (cluster, bulk and layer).

Species	Natom	cluster	layer	bulk	total
Sn16	16	505	3	3154	3662
Sn31	31	0	0	28	28
Sn32	32	0	4	28	32
O1-Sn12	13	0	70	0	70
O1-Sn16	17	0	0	40	40
O2-Sn10	12	0	78	0	78
O2-Sn16	18	0	0	39	39
O4	4	0	94	0	94
O4-Sn8	12	0	0	2080	2080
O4-Sn16	20	0	1	34	35
O4-Sn27	31	0	11	46	57
O6-Sn4	10	0	28	1962	1990
O6-Sn8	14	32	0	0	32
O6-Sn26	32	0	7	38	45
O7-Sn8	15	0	0	2932	2932
O7-Sn16	23	0	4	43	47
O8-Sn6	14	0	9	65	74
O8-Sn8	16	0	0	4239	4239
O10-Sn6	16	0	48	65	113
O10-Sn16	26	0	3	40	43
O11	11	0	478	146	624
O12-Sn8	20	63	34	27	124
O12-Sn16	28	0	3	32	35
O14-Sn7	21	0	28	52	80
O14-Sn8	22	0	1	44	45
O14-Sn16	30	0	15	18	33
O15-Sn8	23	0	1	41	42

O16-Sn8	24	32	34	43	109
O16-Sn16	32	0	79	13	92
O18-Sn7	25	0	31	50	81
O18-Sn8	26	0	31	34	65
O18-Sn9	27	0	39	52	91
O18-Sn12	30	0	2	50	52
O22-Sn16	38	0	0	128	128
O24-Sn16	40	0	0	12	12
total	--	632	1136	15575	17343

Table S2. Structure information in the first principles global dataset of Sn-O-H system. Listed data are the number of the structures in the global dataset, as distinguished by the chemical formula, the number of atoms per cell (Natom), the type of structures (cluster, bulk and layer).

Species	Natom	cluster	layer	bulk	total
Sn16	16	505	3	3154	3662
Sn27	27	0	1	3	4
Sn28	28	0	2	33	35
Sn31	31	0	0	28	28
Sn32	32	0	4	28	32
O1-Sn12	13	0	70	0	70
O1-Sn16	17	0	0	40	40
O1-Sn26	27	0	3	0	3
O1-Sn27	28	0	42	6	48
O2-Sn10	12	0	78	0	78
O2-Sn16	18	0	0	39	39
O2-Sn25	27	0	0	5	5
O2-Sn26	28	0	0	102	102
O3-Sn24	27	0	3	3	6
O3-Sn25	28	0	56	92	148
O4	4	0	94	0	94
O4-Sn8	12	0	0	2080	2080
O4-Sn16	20	0	1	34	35
O4-Sn23	27	0	6	3	9
O4-Sn27	31	0	11	46	57
O5-Sn22	27	0	2	4	6
O5-Sn23	28	0	58	66	124
O6-Sn4	10	0	28	1962	1990
O6-Sn8	14	32	0	0	32
O6-Sn21	27	0	1	1	2
O6-Sn22	28	0	88	49	137
O6-Sn26	32	0	7	38	45

O7-Sn8	15	0	0	2932	2932
O7-Sn16	23	0	4	43	47
O7-Sn20	27	0	8	0	8
O7-Sn21	28	0	77	3	80
O8-Sn6	14	0	9	65	74
O8-Sn8	16	0	0	4239	4239
O8-Sn19	27	0	5	0	5
O8-Sn20	28	0	95	30	125
O10-Sn6	16	0	48	65	113
O10-Sn16	26	0	3	40	43
O10-Sn18	28	0	80	12	92
O11	11	0	478	146	624
O12-Sn8	20	63	34	27	124
O12-Sn15	27	0	5	0	5
O12-Sn16	28	0	104	189	293
O14-Sn7	21	0	28	52	80
O14-Sn8	22	0	1	44	45
O14-Sn14	28	0	40	527	567
O14-Sn16	30	0	15	18	33
O15-Sn8	23	0	1	41	42
O15-Sn13	28	0	2	32	34
O16-Sn8	24	32	34	43	109
O16-Sn16	32	0	79	13	92
O18-Sn7	25	0	31	50	81
O18-Sn8	26	0	31	34	65
O18-Sn9	27	0	39	52	91
O18-Sn12	30	0	2	50	52
O22-Sn16	38	0	0	128	128
O24-Sn12	36	0	201	0	201
O24-Sn16	40	0	0	12	12
H1-Sn15	16	0	0	56	56

H1-O3-Sn32	36	0	8	1	9
H1-O6-Sn29	36	0	3	3	6
H1-O8-Sn4	13	0	27	1401	1428
H1-O8-Sn27	36	0	4	1	5
H1-O11-Sn24	36	0	0	10	10
H1-O12-Sn6	19	0	24	19	43
H1-O15-Sn20	36	0	6	10	16
H1-O18-Sn17	36	0	1	3	4
H1-O20-Sn15	36	0	4	2	6
H1-O23-Sn12	36	0	146	0	146
H2-Sn14	16	0	0	89	89
H2-O4-Sn23	29	0	2	0	2
H2-O6-Sn4	12	0	208	93	301
H2-O7-Sn4	13	0	421	293	714
H2-O8-Sn4	14	37	274	1325	1636
H2-O12-Sn6	20	0	18	8	26
H2-O14-Sn8	24	0	21	0	21
H2-O15-Sn8	25	0	31	0	31
H2-O16-Sn8	26	0	65	140	205
H3-Sn26	29	0	159	16	175
H3-O8-Sn4	15	0	177	446	623
H3-O12-Sn6	21	0	19	16	35
H3-O15-Sn8	26	0	41	0	41
H3-O16-Sn8	27	0	38	0	38
H4-Sn12	16	91	0	52	143
H4-Sn17	21	0	2	27	29
H4-O4-Sn8	16	0	0	20	20
H4-O5-Sn20	29	0	42	0	42
H4-O6-Sn4	14	0	227	118	345
H4-O8-Sn4	16	1	333	316	650
H4-O11-Sn8	23	0	16	35	51

H4-O12-Sn8	24	0	17	55	72
H4-O13-Sn8	25	0	110	50	160
H4-O14-Sn8	26	0	213	32	245
H4-O15-Sn8	27	0	89	6	95
H4-O16-Sn8	28	0	183	48	231
H4-O19-Sn8	31	0	31	0	31
H4-O28-Sn16	48	0	79	0	79
H4-O30-Sn16	50	0	20	170	190
H4-O32-Sn16	52	0	0	200	200
H5-Sn16	21	0	21	1	22
H5-O19-Sn8	32	0	21	0	21
H6-Sn10	16	0	0	40	40
H6-Sn15	21	0	22	0	22
H6-Sn25	31	0	2	0	2
H6-Sn26	32	5	5	10	20
H6-Sn52	58	0	44	7	51
H6-O7-Sn16	29	0	12	14	26
H6-O10-Sn8	24	0	21	16	37
H6-O15-Sn8	29	0	15	44	59
H6-O16-Sn8	30	0	41	16	57
H6-O19-Sn8	33	0	28	0	28
H6-O28-Sn16	50	0	53	130	183
H6-O30-Sn16	52	0	138	0	138
H6-O32-Sn16	54	0	142	0	142
H7-Sn25	32	0	0	1	1
H7-O16-Sn8	31	0	39	0	39
H8-Sn8	16	94	2	165	261
H8-O8-Sn8	24	0	37	18	55
H8-O10-Sn40	58	0	95	0	95
H8-O11-Sn8	27	0	51	36	87
H8-O12-Sn8	28	0	132	11	143

H8-O15-Sn8	31	0	34	26	60
H8-O16-Sn8	32	0	45	37	82
H8-O19-Sn8	35	0	2	1	3
H8-O22-Sn16	46	0	106	0	106
H8-O26-Sn16	50	0	322	214	536
H8-O30-Sn16	54	0	175	136	311
H8-O38-Sn16	62	0	115	0	115
H9-Sn23	32	1	0	0	1
H9-O13-Sn8	30	0	236	0	236
H10-Sn21	31	0	3	0	3
H10-Sn22	32	0	1	12	13
H10-O1-Sn18	29	0	40	0	40
H10-O10-Sn8	28	0	104	189	293
H10-O12-Sn8	30	0	117	104	221
H10-O13-Sn6	29	0	118	84	202
H10-O13-Sn7	30	0	182	0	182
H10-O13-Sn8	31	0	83	125	208
H10-O20-Sn8	38	0	6	2	8
H10-O21-Sn8	39	0	5	1	6
H10-O38-Sn16	64	0	95	0	95
H11-Sn5	16	0	0	92	92
H11-Sn18	29	0	94	6	100
H11-Sn21	32	0	0	3	3
H11-O12-Sn8	31	0	203	0	203
H12-O14-Sn32	58	0	80	14	94
H12-O20-Sn16	48	0	143	62	205
H12-O30-Sn16	58	0	46	128	174

H12-O38-Sn16	66	0	118	0	118
H13-Sn19	32	0	0	6	6
H14-Sn18	32	0	0	3	3
H14-O7	21	0	1	808	809
H14-O21-Sn8	43	0	3	1	4
H14-O32-Sn16	62	0	90	0	90
H15-Sn6	21	0	58	0	58
H15-Sn16	31	0	0	4	4
H15-Sn17	32	0	0	13	13
H16-Sn5	21	0	68	0	68
H16-Sn16	32	0	0	24	24
H16-O8	24	0	14	3955	3969
H16-O16-Sn16	48	0	118	7	125
H16-O22-Sn16	54	0	115	1	116
H16-O30-Sn16	62	0	62	111	173
H17-Sn15	32	0	0	2	2
H18-Sn13	31	0	0	2	2
H19-Sn13	32	0	0	3	3
H20-O2-Sn36	58	0	91	0	91
H22-Sn30	52	0	58	6	64
H22-Sn36	58	0	47	6	53
H23-Sn8	31	0	3	1	4
H23-Sn9	32	0	5	6	11
H24-Sn8	32	0	0	3	3
H30-O15	45	124	4	94	222
H32-O38-	82	0	43	173	216

Sn12					
H32-O39-Sn12	83	0	68	139	207
H32-O40-Sn12	84	0	52	122	174
H36-O43-Sn15	94	0	25	0	25
H38-O47-Sn16	101	0	40	0	40
H39-O47-Sn15	101	0	32	0	32
H39-O50-Sn16	105	0	190	1	191
H40-O47-Sn16	103	0	78	1	79
H41-O49-Sn16	106	0	117	14	131
H42-O46-Sn15	103	0	23	0	23
H42-O50-Sn16	108	0	136	2	138
H104-O114-Sn32	250	0	7	47	54
H104-O116-Sn32	252	0	26	55	81
total	--	985	10039	29119	40143

Table S3. Comparison of adsorption energies for different functionals. All values are given in eV.

**Adsorbed species**	**ΔE_{ads}_PBE**	**ΔE_{ads}_PBE+D3**	**$\Delta\Delta E$**
HCOO*	-0.67	-0.89	-0.22
*COOH	0.41	0.19	-0.22
H*	0.23	0.18	-0.05
CO_2	-0.02	-0.10	-0.09
CO	-0.01	-0.10	-0.09

Table S4. The zero-point energy correction, enthalpy correction, and entropy correction for adsorbates and free molecules. All values are given in eV.

**Adsorbates**	**ZPE**	**δH_0**	**TS**
HCOO*	0.64	0.11	0.22
*COOH	0.58	0.08	0.17
H*	0.16	0.01	0.01
CO_2	0.31	0.11	0.66
CO	0.14	0.10	0.62
HCOOH	0.90	0.11	1.02
H_2O	0.58	0.10	0.66
H_2	0.28	0.09	0.40

Table S5. The iCOHP of HCOO* on SnO_x surfaces with different reduction degree. All values are given in eV.

Surfaces	Sn_1-O_1	Sn_2-O_2	Sum
$1O_{v\_d}$	–3.18	–3.18	–6.36
$2O_{v\_d}$	–2.77	–2.87	–5.64
$3O_{v\_d}$	–2.06	–2.41	–4.47
$4O_{v\_d}$	–1.57	–1.78	–3.35

Table S6. The iCOHP of *COOH on SnO_x surfaces with different reduction degree. All values are given in eV.

Surfaces	Sn-C
$1O_{v\_d}$	–4.14
$2O_{v\_d}$	–4.12
$3O_{v\_d}$	–3.90
$4O_{v\_d}$	–3.03

Table S7. The iCOHP of H* on SnO_x surfaces with different reduction degree. All values are given in eV.

Surfaces	Sn_1-H	Sn_2-H	Sum
$1O_{v\_d}$	–2.42	–2.44	–4.87
$2O_{v\_d}$	–2.56	–2.29	–4.85
$3O_{v\_d}$	–2.31	–2.36	–4.67
$4O_{v\_d}$	–2.32	–2.23	–4.55

Table S8. Periodic NBO analyses of Sn-O bonds between HCOO* and tin oxide surfaces.

Active Site	NBO	Occupancy	Hybridization (%)
$1O_{v\_d}$	Sn-O (σ*)	0.20	O: 11.18, Sn: 88.92
	Sn-O (σ)	1.90	O: 88.92, Sn: 11.18
$2O_{v\_d}$	Sn-O (σ*)	0.46	O: 12.45, Sn: 87.55
	Sn-O (σ)	1.92	O: 87.55, Sn: 12.45
$3O_{v\_d}$	Sn-O (σ*)	0.58	O: 9.27, Sn: 90.73
	Sn-O (σ)	1.88	O: 90.73, Sn: 9.27
$4O_{v\_d}$	Sn-O (σ*)	0.88	O: 12.64, Sn: 87.36
	Sn-O (σ)	1.90	O: 87.36, Sn: 12.64

Table S9. Periodic NBO analyses of Sn-C bonds between *COOH and tin oxide surfaces.

**Active Site**	**NBO**	**Occupancy**	**Hybridization (%)**
$1O_{v\_d}$	Sn-C (σ*)	0.12	Sn: 77.14, C: 22.86
	Sn-C (σ)	1.80	Sn: 22.86, C: 77.14
$2O_{v\_d}$	Sn-C (σ*)	0.16	Sn: 72.79, C: 27.21
	Sn-C (σ)	1.81	Sn: 27.21, C: 72.79
$3O_{v\_d}$	Sn-C (σ*)	0.28	Sn: 63.37, C: 36.63
	Sn-C (σ)	1.83	Sn: 36.63, C: 63.37
$4O_{v\_d}$	Sn-C (σ*)	0.52	Sn: 60.73, C: 39.27
	Sn-C (σ)	1.81	Sn: 39.27, C: 39.27

References

[1] L. Li, Z.-J. Zhao, C. Hu et al., "Tuning Oxygen Vacancies of Oxides to Promote Electrocatalytic Reduction of Carbon Dioxide," ACS Energy Letters, vol. 5, no. 2, pp. 552-558, 2020.

[2] F. Dattila, R. García-Muelas and N. López, "Active and Selective Ensembles in Oxide-Derived Copper Catalysts for CO_2 Reduction," ACS Energy Letters, pp. 3176-3184, 2020.

[3] T. Eom, W. J. Kim, H.-K. Lim et al., "Cluster Expansion Method for Simulating Realistic Size of Nanoparticle Catalysts with an Application in CO_2 Electroreduction," The Journal of Physical Chemistry C, vol. 122, no. 16, pp. 9245-9254, 2018.

[4] C. Hu, L. Li, W. Deng et al., "Selective Electroreduction of Carbon Dioxide over SnO_2-Nanodot Catalysts," ChemSusChem, vol. 13, pp. 1-8, 2020.

[5] T. Xie and J. C. Grossman, "Crystal Graph Convolutional Neural Networks for an Accurate and Interpretable Prediction of Material Properties," Physical Review Letters, vol. 120, no. 14, pp. 145301, 2018.

[6] M. He, B. Xu and Q. Lu, "Probing the role of surface speciation of tin oxide and tin catalysts on CO_2 electroreduction combining in situ Raman spectroscopy and reactivity investigations," Chinese Journal of Catalysis, vol. 43, no. 6, pp. 1473-1477, 2022.

[7] Y. W. Choi, F. Scholten, I. Sinev and B. Roldan Cuenya, "Enhanced Stability and CO/Formate Selectivity of Plasma-Treated SnO_x/AgO_x Catalysts during CO_2 Electroreduction," Journal of the American Chemical Society, vol. 141, no. 13, pp. 5261-5266, 2019.
